# A comprehensive framework for evaluation of high pacing frequency and arrhythmic optical mapping signals

**DOI:** 10.3389/fphys.2023.734356

**Published:** 2023-01-23

**Authors:** Girish S. Ramlugun, Kanchan Kulkarni, Nestor Pallares-Lupon, Bastiaan J. Boukens, Igor R. Efimov, Edward J. Vigmond, Olivier Bernus, Richard D. Walton

**Affiliations:** ^1^ IHU-Liryc, Fondation Bordeaux Université, Pessac-Bordeaux, France; ^2^ Univ. Bordeaux, Inserm, Centre de Recherche Cardio-Thoracique, Bordeaux, France; ^3^ Department of Physiology, Cardiovascular Research Institute Maastricht, University Maastricht, Maastricht, Netherlands; ^4^ Department of Medical Biology, Amsterdam Cardiovascular Sciences, Amsterdam University Medical Center, University of Amsterdam, Amsterdam, Netherlands; ^5^ Department of Biomedical Engineering, The George Washington University, Washington, DC, United States; ^6^ Department of Biomedical Engineering, Northwestern University, Chicago, IL, United States; ^7^ Department of Medicine, Northwestern University, Chicago, IL, United States; ^8^ Univ. Bordeaux, Centre National de la Recherche Scientifique (CNRS), Institut de Mathématiques de Bordeaux, UMR5251, Bordeaux, France

**Keywords:** optical mapping, fibrillation, pacing, electrophysiology, image processing

## Abstract

**Introduction:** High pacing frequency or irregular activity due to arrhythmia produces complex optical mapping signals and challenges for processing. The objective is to establish an automated activation time-based analytical framework applicable to optical mapping images of complex electrical behavior.

**Methods:** Optical mapping signals with varying complexity from sheep (*N* = 7) ventricular preparations were examined. Windows of activation centered on each action potential upstroke were derived using Hilbert transform phase. Upstroke morphology was evaluated for potential multiple activation components and peaks of upstroke signal derivatives defined activation time. Spatially and temporally clustered activation time points were grouped in to wave fronts for individual processing. Each activation time point was evaluated for corresponding repolarization times. Each wave front was subsequently classified based on repetitive or non-repetitive events. Wave fronts were evaluated for activation time minima defining sites of wave front origin. A visualization tool was further developed to probe dynamically the ensemble activation sequence.

**Results:** Our framework facilitated activation time mapping during complex dynamic events including transitions to rotor-like reentry and ventricular fibrillation. We showed that using fixed AT windows to extract AT maps can impair interpretation of the activation sequence. However, the phase windowing of action potential upstrokes enabled accurate recapitulation of repetitive behavior, providing spatially coherent activation patterns. We further demonstrate that grouping the spatio-temporal distribution of AT points in to coherent wave fronts, facilitated interpretation of isolated conduction events, such as conduction slowing, and to derive dynamic changes in repolarization properties. Focal origins precisely detected sites of stimulation origin and breakthrough for individual wave fronts. Furthermore, a visualization tool to dynamically probe activation time windows during reentry revealed a critical single static line of conduction slowing associated with the rotation core.

**Conclusion:** This comprehensive analytical framework enables detailed quantitative assessment and visualization of complex electrical behavior.

## 1 Introduction

Chaotic electrical activity in the ventricles of the heart form the precursor to sudden cardiac death, a major public health problem. Our understanding of arrhythmic behavior and electrical remodeling in cardiac diseases at the organ level continues to grow with advancements of recording instruments. Optical mapping using potentiometric fluorescent probes excels in the compromise over resolution and imaging field of view (Himel and Knisley, 2007). Yet, spatio-temporal complexity of electrical signals recorded during chaotic electrical events such as in cardiac arrhythmias, imposes constraints to accurately interpret the electrical behavior. Increased frequency of activity often leads to concomitant slowing of overall conduction, increased variance of conduction velocities and a loss of synchronization (Kleber et al., 1986; Akar et al., 2007); the latter, impacting negatively the signal amplitude (Fast and Kleber, 1995). Moreover, multiple complex arrhythmic patterns may co-exist, originate from numerous sources and transition from one behavior to another over time. As a result, algorithmic activation time (AT) detection, visualization and interpretation are challenging and a unified approach encompassing a broad range of signal complexities is lacking.

The first 20 s following the induction of ventricular fibrillation in pig showed chaotic electrical impulse propagation evolving towards steadily more organized states (Rogers et al., 1997a; Rogers et al., 1997b). To understand and quantify waveform complexity, a multiplicity metric was developed to determine the repetitiveness of propagating wave fronts, which are dependent on their orientation, size and regularity. Chen et al. (2000) later showed using frequency analysis and phase mapping that regions of the ventricles in rabbit maintained periodic activity at frequencies exceeding the surrounding tissue. From this, they determined that high frequency periodic sources were responsible for driving complex fibrillatory behavior in the periphery, underlying ventricular fibrillation. However, the life-time of rotors when measuring from the epicardial surface are variable and rarely lasting more than two rotations in healthy myocardium (Kay et al., 2006). In this case, rotors were identified as the point of phase singularity, where contours of all phases converge to form the center of rotation (Winfree, 1989). More recently, it was demonstrated that the point of phase convergence could also assume a line, which aligned with functional gradients or structural boundaries (Arno et al., 2021). The wavelet hypothesis was evaluated using *in silico* models and suggested that multiple wavelets could be sustained in homogeneous tissue, but preferentially block or lead to wave break with increased heterogeneity (Fenton et al., 2002). Short-lived and intermittent renewal of rotor sources or the constant wave break and regeneration of wavelets describe at least part of the fundamental complexity of fibrillatory behavior. But this is further confounded in tissue by structural heterogeneities, particularly in pathological conditions resulting in increased electrical uncoupling.

Phase analysis of electrograms and optical mapping studies of tachyarrhythmic events has proven effective at identifying the substrate region implicated in classical rotor theory, but is less adapted currently for applications to assess macroreentry or non-reentrant events. Frequency analysis requires multiple electrical events to determine the periodicity and regularity of activity. However there remains an unmet need to provide a unified approach to spatially resolve electrical propagation patterns and to investigate wave front behavior in an individualized manner, irrespective of the underlying mechanism.

AT mapping has been used successfully to track critical excitatory pathways underlying stable ventricular tachycardia (Takahashi et al., 2004). Child et al. (2015) developed the reentry vulnerability index from an activation-repolarization time metric between the proximal and distal ends of the same wave front. Requiring only a short-coupled stimulation protocol, the vulnerability index provided maps of the relative probability of reentry without needing to induce the arrhythmia. Focal sources of activation can also be readily located as local sites of AT minima. Although surface mapping methods cannot distinguish between focal sources originating from the mapped surface or deeper layers and are therefore termed sites of activation breakthrough. That being said, optical mapping signals originate from a near-surface tissue volume and are therefore an integral of electrical responses from several cell layers (Fedorov et al., 2010). As a result, optical action potential upstroke morphology depends on the sequence that the different cell layers are activated and provides an approximation of the subsurface electrical wave orientation (Hyatt et al., 2008). Moreover, the depth contribution of optical signals can be varied by modifying the wavelength of excitation light (Walton et al., 2010); A palette of voltage-sensitive dyes extending in to the near-infrared range continue to be developed (Matiukas et al., 2007). The versatility, effective resolution, signal morphology and additional depth information afforded by optical mapping necessitates a dedicated framework for analysis of complex tachyarrhythmia.

The objective of this study was to establish a novel analytical framework for assessing electrical complexity recorded by optical mapping. Specifically, aims were to: Conserve image resolution; avoid temporal signal complexity reduction; conserve multiple upstroke events; To achieve spatial coherence of AT; To perform classification of repetitive/non-repetitive activity and to spatially classify wave fronts by their source. We present a comprehensive pipeline based on a novel unified method to assess the activation sequence adapted for arrhythmic and transitional electrical behavior. We compared experimental recordings with an *in silico* model of reentry induced in a three-dimensional ventricular slab geometry. Optical mapping signals were derived from the *in silico* model to apply processing and analyses on signals with appropriate morphology, consistent with optical mapping experiments (Hyatt et al., 2005; Walton et al., 2012).

## 2 Methods and materials

### 2.1 *In silico* model

A finite element model of a three-dimensional ventricular wedge, measuring 5 cm × 5 cm in the epicardial plane and 1 cm transmural thickness, was created at a spatial resolution of 200 µm isotropic. Fibre orientation was set to vary by 120from the endocardial to the epicardial surfaces. The Ten Tusscher ionic model (Ten Tusscher and Panfilov, 2006) was implemented to simulate ventricular arrhythmia. Monodomain simulations were performed using the openCARP simulation environment (Plank et al., 2021) with baseline conductivity values of 0.03, 0.02 and 0.02 S/m for longitudinal (*σ*_L_), transverse (*σ*_T_) and transmural (*σ*_S_) directions. Using time steps of 0.05 ms throughout, a planar wave was generated by stimulating one transmural surface of the slab with shortening coupling intervals at 0, 325, 525 and 715 ms. A rotating wave was then generated by applying a cross field stimulus during the recovery phase of the central portion of the model at 860 ms (Skouibine et al., 2002). To emulate experiments and signal morphology specific to optical mapping, the cardiac arrhythmia research package (Vigmond et al., 2003) was used to generate epi-fluorescent optical signals from electrical simulations, as described previously (Bishop et al., 2006).

### 2.2 Tissue acquisition

Hearts were obtained from sheep (N = 8, 2 years old) weighing 40–50 kg in accordance with the guidelines from Directive 2010/63/EU of the European Parliament on the protection of animals used for scientific purposes and the local ethical committee. Healthy sheep (*N* = 7) and a sheep (*N* = 1) with chronic myocardial infarction following coronary embolization (see Pallares-Lupon et al., 2022) were premedicated with ketamine (20 mg/kg) and acepromazine (0.02 mL/kg), anesthesia was induced by propofol (2 mg/kg) and maintained under isoflurane, 2%, in air/O2 (50/50%) after intratracheal intubation. Sheep were euthanized by intravenous injection with pentobarbital (30 mL/50 Kg) and hearts were rapidly excised, cannulated and flushed with cardioplegic solution, containing (mM): NaCl, 110; CaCl2, 1.2; KCl, 16; MgCL2, 16; NaHCO3, 10 and glucose, 9.01 at 4°C.

### 2.3 Preparations of sheep myocardium

Coronary-perfused ventricular wedges were prepared by dissection in to two different configurations, based on the major coronary artery perfused: right ventricle (right coronary artery) and left ventricle (left anterior descending and circumflex arteries (Moreno et al., 2019)). In each case, cannulation of the coronary circulation was applied at the ostia, arising from the aortic root. Perfusion leaks at cut surfaces were carefully tied-off and preparations mounted on to a frame, exposing the endocardial surfaces. Wedges were submersed and perfused with a saline solution gassed with 95%/5% O2/CO2 and containing (mM): NaCl, 130; NaHCO3, 24; NH2PO4, 1.2; MgCl2, 1; glucose, 5.6; KCl, 4; CaCl2, 1.8, at 37°C and pH7.4.

### 2.4 Optical mapping

Preparations were imaged using optical mapping of the endocardial surfaces after being mechanically-uncoupled using 15 µM blebbistatin, and loaded with the voltage-sensitive dye, Di-4-ANEPPS (Cytocybernetics, United States). Endocardial surfaces were illuminated with monochromatic LEDs at 530 nm (Cairn Research Ltd, Kent, United Kingdom). Optical images (100 × 100 pixels) of signals passed through a 650 ± 20 nm band-pass filter were acquired using a Micam Ultima CMOS camera (SciMedia United States Ltd, CA, United States) at 1 kHz with a spatial resolutions of 0.7 × 0.7 mm.

### 2.5 Pacing protocols

**FIGURE 1:**
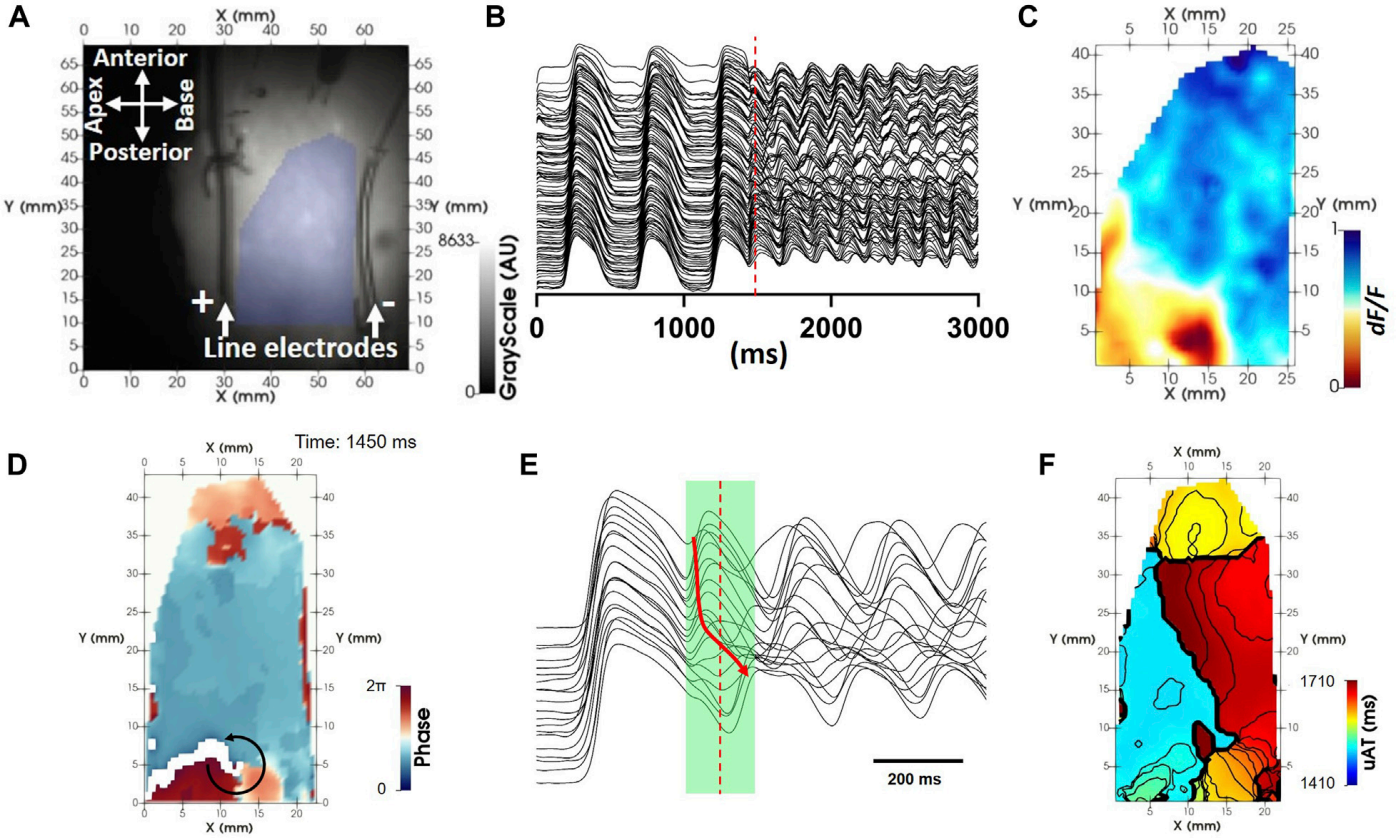
uAT mapping extracted from a fixed time window during reentry. **(A)** Background optical mapping image of the endocardial surface from a coronary-perfused right ventricle. The region of interest lying in between two line electrodes for reentry induction is highlighted by blue shading. **(B)** Example pre-processed optical mapping signals extracted from the full region of interest. **(C)** A snapshot of the dynamic voltage-dependent optical signal (*F/dF*) during reentry [time indicated by the red dashed line in **(B)**]. **(D)** An instantaneous phase map derived using the Hilbert transform showing evidence for a repetitive propagating wave front [red dashed line in **(B)**]. **(E)** Example pre-processed optical mapping signals extracted from pixels along the circular black arrow in **(D)**. A green shaded region indicates a uniform time window used to derive uAT points using the uniform window method for calculation of activation time. The red arrow shows the principle sequence of activation. **(F)** uAT map corresponding to the fixed time window shown in B during reentry. Isolines are spaced 10 ms.

To demonstrate the analysis pipeline, an optical mapping acquisition for the induction of reentry composed of a single rotor was recorded from the right ventricle. Figure 1A shows two parallel 4 cm platinum line electrodes (Cardialen Inc, United States) were sutured to the endocardial surface at the atrioventricular ring and along the apico-anterior border of the preparation (approximately 5 cm apart). The preparation was paced by a train of pulses at twice the threshold at a fixed frequency of 2 Hz from the posterior border. Depolarization and repolarization fronts were near planar in between, and perpendicular to the line electrodes. With a delay of 220 ms from the last planar stimulation pulse, an electrical cross-field shock at 80 V was applied between the parallel electrodes, creating a voltage gradient of 16 V/cm. This was found to fall in the critical window of repolarization for induction of self-sustaining arrhythmic activity by the cross-field shock-on-T wave mechanism (Frazier et al., 1989) (Figure 1B). Following tachyarrhythmia induction, pacing was discontinued.

To observe transitioning dynamic electrical behavior from basal pacing rates to ventricular arrhythmia, left ventricles from a set of 5 sheep were optically mapped during increasingly shortened coupling intervals of pacing (S1S2S3S4 protocol). Following a stimulus train of ten beats (S1) at a frequency of 1.5 Hz, short coupled pulses were delivered just above the effective refractory period (ERP), with a precision of 5 ms. The ERP was determined by testing stimulation responses of tissue at decreasing coupling intervals starting from the interval of the preceding stimulation pulse. Therefore, S2 responses were tested for the following intervals (ms): 667, 600, 500, 450, 430, 410, 400, 390, …, decreasing by 10 ms until loss of capture. Intervals would then be stepped up 5 ms at a time until capture was reestablished. The pacing regime was continued, until a maximum S4 short-coupled stimuli and either ventricular fibrillation was initiated or a loss of capture.

### 2.6 Signal processing methods

All pre-conditioning and post-processing procedures were performed using custom-built software developed in our lab using the programming environment PV wave. Each specific processing and analytical procedure described below is detailed using universal pseudocode in the Supplementary Material.

### 2.7 Pre-conditioning of optical mapping signals

We consider optical mapping data as a time-series containing T image matrices, each with dimensions *X*, *Y*. Prior to initiating the analysis pipeline described herein, all voltage-sensitive fluorescent signals (F) first underwent filtering using a forwards-backwards butterworth digital filter with a low-pass cut-off at 60 Hz, spatial averaging using a 3 × 3 pixel uniform average filter and a 3-frame uniform running-average temporal filter. Signals were inverted and the magnitude of fluorescent changes normalized from 0 to 1 in each pixel (Figure 1C). A region of interest (Figure 1A) was defined for each experiment to identify foreground pixels and exclude background pixels containing noise (set to zero).

The tachyarrhythmia induced by cross-field shock-on-T example (Figure 1B) is used to illustrate the signal processing pipeline proposed herein. Phase mapping of the tachyarrhythmia showed a circuitous activation pattern (Figure 1D; Supplementary Video S1), which appeared to repeat over several consecutive APs (Figure 1E). ATs were sought from optical mapping signals during the arrhythmic episode using a uniform time window across all pixels (green shaded region in Figure 1E) for comparison with the proposed novel approach. ATs derived from a uniform window, uAT, failed to recapitulate a circuitous activation sequence (Figure 1F). The uniform time window was manually validated to incorporate the beginning of the earliest AP upstroke and the end (peak) of the latest upstroke during a single reentrant cycle.

### 2.8 Novel post-processing pipeline for analysis of complex optical mapping signals

**FIGURE 2:**
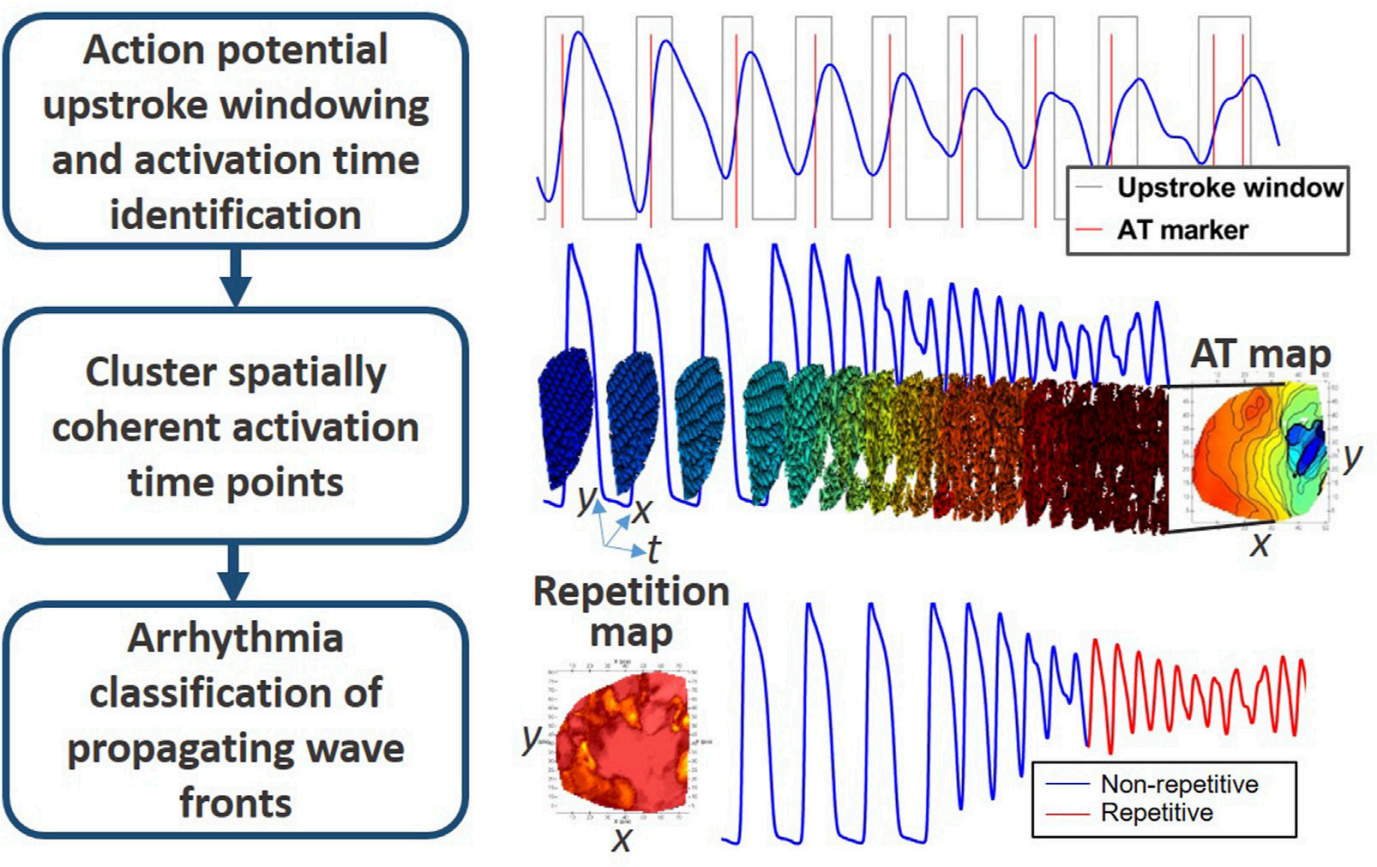
Schematic workflow of the novel pAT mapping framework.

The proposed method herein is a novel framework for AT-based processing and analysis of complex signals. The comprehensive pipeline is summarized in Figure 2 in to three main sections: 1) Deriving AT; 2) Associating AT points to common wave fronts and 3) classification and quantification of electrical behavior.

#### 2.8.1 Deriving AT

##### 2.8.1.1 Pixel-independent optical action potential upstroke windowing

**FIGURE 3:**
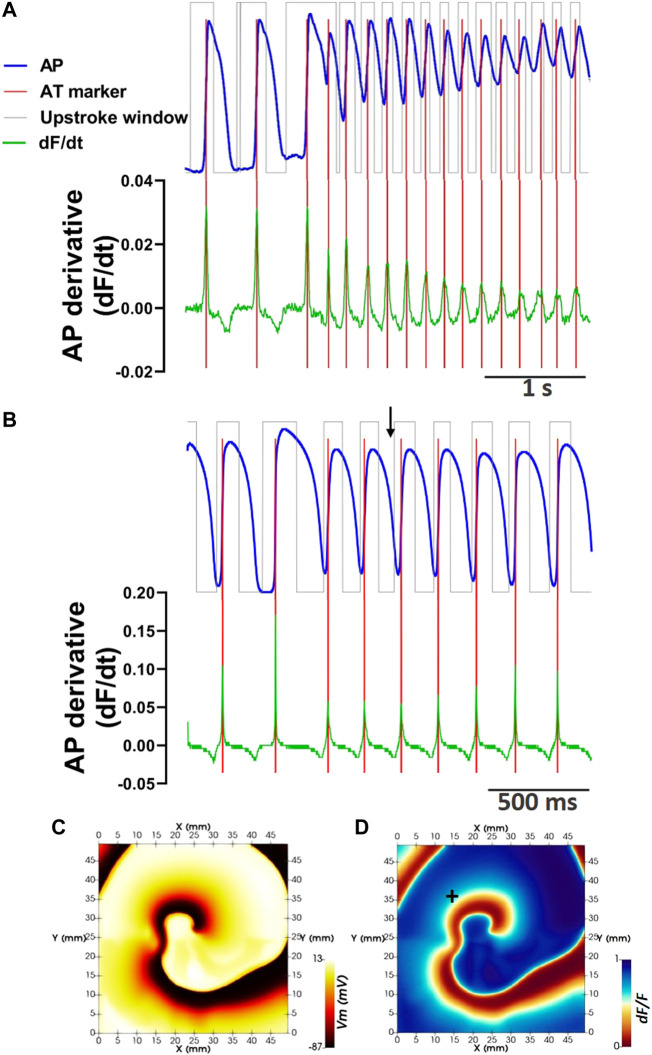
Defining phase window-independent upstroke morphology and pAT using the signal derivative. **(A)** Extraction of the pre-processed optical action potential signal during reentry from pixel coordinate (20,10). Corresponding phase windows of pAT are superimposed. The derivative of the corresponding signal is shown. Pronounced derivative peaks falling within phase windows indicate pAT (red lines). **(B)** Similarly, an optical action potential signal and corresponding derivative extracted from an *in silico* model of reentry. **(C)** Membrane voltage (*Vm*) map extracted from the epicardial surface of the electrical *in silico* model at the time indicated by a black arrow in **(B)**. **(D)** Corresponding *F/dF* computed from the *in silico* model. In silico optical signal in B extracted from pixel indicated by **+**.

Time windows centered on action potential upstrokes were sought in a non-uniform, pixel-independent manner. All signals were temporarily mean-subtracted for computation of phase (*φ*) using a Hilbert Transform, with a negative phase-shift of 90°. Considering *φ* ranges from 0 to 2 π, taking *φ*>*π*, provided positive deflections of *φ* coinciding with, and encompassing action potential upstrokes from an experimental recording (Figure 3A) and a simulation of myocardial reentry (Figure 3B). Deriving *φ* using the Hilbert Transform is robust for periodic signals but fails when signals are non-periodic. Therefore, phase computation was applied to signal segments (Supplementary Figure S1). Further splitting of segments and phase computation was applied iteratively until segments reached a minimum duration of 128 ms. The resultant phase of the full-length signal was determined by assigning a value of 2π at all instances where *φ*>*π* was observed in any corresponding segments. To avoid overpopulation of false positive phase detection, a second filtering step was applied. Windows where φ = 2π were rejected if the corresponding optical signals showed linear regression ≤0 or a maximal derivative below a predefined derivative threshold (θ(*dF*⁄*dt*)), defined either manually, or through automation (Section 2.8.1.2 Automated definition of a signal derivative threshold for AT). The time of maximal derivative of fluorescent signals during each phase-derived upstroke window defined AT using the novel method (pAT).

##### 2.8.1.2 Automated definition of a signal derivative threshold for AT

Derivatives of optical mapping signals are highly susceptible to influences by noise. Thus a minimum, *θ*_*dF*⁄*dt*_ (>0) should be assigned to increase the probability of detecting true depolarizing events. Either a user-defined threshold can be employed based on the impact of background noise or alternatively, we propose an automated approach that approximates a single derivative threshold value that can be applied across all unmasked pixels and upstroke windows. The maximal derivative for each phase-derived time window from each pixel were identified. To reduce the influence of noise, outliers of maximum derivative values were removed using the False Discovery Rate method (Motulsky and Brown, 2006), where θ′_*dF*⁄*dt*_ equals the maximum desired false discovery rate of 1%.

##### 2.8.1.3 Defining AT

The fundamental approach of defining pAT by the maximal derivative is the same as described for conventional methods (Walton et al., 2012), however we apply two additional constraints. These are: 1) That ATs are sought on a pixel-independent and upstroke window-independent basis and 2) Upstroke morphology is evaluated to identify a maximum of two AT points per upstroke. For the latter, Upstroke morphology was characterized from derivative profiles of the upstroke signal. The two largest first-order derivative maxima exceeding θ′_*dF*⁄*dt*_ were considered potential AT points (Figures 3A, B). If two potential AT points were detected, the signal derivative was further evaluated to determine of the derivative maxima are attributed to the same or separate depolarization events. The valley depth of the signal derivative between the two largest derivative maxima was measured relative to the amplitude of the smaller of the two maxima. The current study assumed a minimum valley depth of 75% to consider the upstroke morphology to be the result of two independent activation events (Fedorov et al., 2010). Otherwise, only the time of the largest derivative maxima was determined to be a pAT.

To store and manipulate pAT values, a new pAT matrix with dimensions *X*,*Y*,*T* was considered. For each pixel and upstroke window pAT values were soughtafter as described above and the pAT matrix was updated in the corresponding pixel (*x*,*y*) and frame corresponding to pAT, *t*, with a value of 1.

#### 2.8.2 Associating pAT points to common wave fronts

**FIGURE 4:**
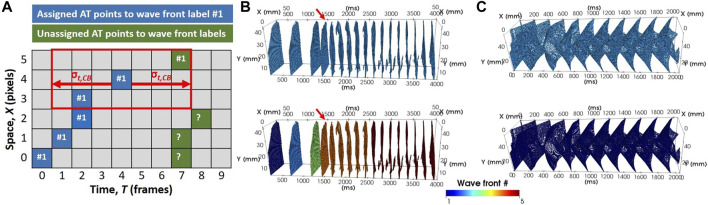
Adapted connected components algorithm for wave front clustering. **(A)** A schematic diagram illustrates pAT points on a space-time plot. In the example, wave front label assignment has been made for all pAT points in the first 4 frames to label #1. Let the current pAT point be evaluated for connectivity be at pixel 4, frame 4 on the space-time plot. The red box illustrates the space-time bounds of eligible connectivity. In this case, the currently unassigned pAT point at pixel 5, frame 7 lies within the connectivity boundary and will be assigned to wave front label #1. **(B)** A spatio-temporal distribution of pAT points for the first 4 s of pacing and reentry induction in experiments (upper panel). pAT points are assigned colors based on their wave front label assignment (lower panel). **(C)** Similarly, pAT points of the *in silico* model from the moment of reentry induction (upper panel) and wave front label assignment (lower panel).

Wave fronts were identified by grouping spatially and temporally associated pAT points in the pAT matrix using an adapted connected components algorithm. A wave front was defined as a single object composed of all propagating fronts that spatially and temporally converge. With this definition, a single wave front therefore may originate from multiple source locations. Connectivity within the pAT matrix is assessed by iterating through pAT points. Connectivity in the spatial domain only considered the immediate neighboring pixels in an 8-point neighborhood. However, wave front propagation from one pixel to its neighbor can be susceptible to conduction delays far exceeding a single time frame (Figure 4A). This required determining the maximum pAT gradient that could be considered as slowed but successful impulse propagation versus conduction block. A threshold of the delay defining conduction block (*θ*_*t,CB*_) may be a user-defined parameter, based on known literature of the species and conditions applied. However, we propose an alternative method to compute *θ*_*t,CB*_ automatically based on real experimental conditions and individual behavior of the tissue. First we describe how *θ*_*t,CB*_ is obtained automatically, followed by the procedure to derive adaptive connected component labels of individual wave fronts.

##### 2.8.2.1 Automated definition of the conduction block parameter

The automated conduction block parameter is defined using an experiment-specific reference acquisition during progressively shortened stimuli (Section 2.5 Pacing protocols). An automated conduction block parameter, *θ*_*t,CB*_, assesses local conduction delays in an 8-point neighborhood. The parameter *θ*_*t,CB*_ was defined as the maximum conduction delay (to the nearest 1 ms) observed with a number of occurrences equal to the number of short-coupled stimuli applied (i.e., for a S1S2S3S4 stimulation protocol, *θ*_*t,CB*_ was defined as the largest common activation gradient observed between four pixel pairs during all stimuli.

##### 2.8.2.2 Connected components labelling of wave fronts

A connected components labelling scheme was applied to the pAT matrix (Figure 4). The following iterative procedure was applied incrementally (looping fastest through *X*, then *Y*, then *T*) across all detected pAT points.

1) Assign label value 1 to the element corresponding to the first indexed pAT point.
2) Let the current pAT point be found at (*x*, *y*, *t*). Evaluate connectivity with adjacent unlabeled pAT points in a local 8-point spatial neighborhood and over the time interval extending from t–*θ*_*t,CB*_ to t + *θ*_*t,CB*_ *.* In the absence of unassigned pAT points, go directly to (3). If pAT points not yet assigned a label exist, assign the same label value as *pAT* (*x,y,t*), and add the neighboring pAT point coordinates for matrix *X*,*Y*,*T* as the first elements in a queue; then go to (3).
3) Remove the original indexed pAT element from the queue and repeat (2) until there are no more elements in the queue. Go to (4).
4) Increment label by 1. By looping fastest through *X*, then *Y*, then *T* to the next unassigned pAT point of the pAT matrix; then go to (2).

#### 2.8.3 Characterisation and classification of electrical behavior

##### 2.8.3.1 Mapping wave front pAT

Each wave front identified by their unique label number was assessed independently for activation and repolarization distribution characteristics. Activation time maps of dimensions X,Y were derived for individual wave fronts. Iterating through each pixel, the earliest pAT point with the corresponding wave front label was plotted on the wave front label AT map. In cases where biphasic upstroke morphologies identified a second short-coupled pAT point (Section 2.8.1.3 Defining pAT), a second wave front label AT map was created to conserve information regarding local disassociated conduction patterns.

##### 2.8.3.2 Mapping wave front repolarization characteristics

Repolarization time (RT) was similarly identified in a pixel- and label-wise manner. From any given pAT, the corresponding optical action potential was evaluated for the signal’s recovery from excitation. The RT is measured from a user-defined percentage drop in signal amplitude from the action potential peak (for example, 80% of repolarization). The time window to search for RT was constrained to APD limits (*AP*D*’*min and *A*PD’max) measured from the earliest pAT of the corresponding action potential. Provisionally, limits used were user-selected for suitability to the species, state and experimental conditions implemented. However, for any individual wave front, a second set of minimum and maximum action potential duration limits (APD”min and *A*PD”ma*x*) were established for the *n* ^th^ pAT. Universal psuedocode is provided in the supplemental materials detailing *A*PD”min and *A*PD”max definitions. Within the refined APD limits the RT was determined based on the normalized signal amplitude of the full analyzed time window. It should be noted however that baseline elevation is a common occurrence when electrical activity becomes increasingly dyssynchronous at a local level. Therefore, signals during episodes of ventricular fibrillation, and particularly those capturing a transition from stable rhythm to fibrillatory behavior may not necessarily repolarize to the same baseline as signals during non-arrhythmic events. Therefore signals during arrhythmia initiation may have dynamic baselines, rendering the true level of repolarization challenging to define. For such cases, an alternative definition of RT is proposed based on the time of minimum signal derivative (Salama et al., 1989; Salama et al., 1994). Similar to pAT, RTs for each wave front were projected to maps *RT* [*X,Y*]. Corresponding APD maps were subsequently derived by subtracting pAT from the RT of corresponding pixels.

##### 2.8.3.3 Classification of repetitive and non-repetitive wave fronts

Wave fronts were assessed for repeated excitation of the same regions of tissue over two or more cycles and wave front labels were classified as either repetitive or non-repetitive, accordingly. Firstly, each pixel of a given wave front was assessed for incidences of pAT repetition. If a majority of pixels for a given wave front had repetitiveness of pAT points, then this wave front was designated with a repetition classification. This classification scheme imposed the following criteria: A minimum cycle length between repetitive excitation events, defined by *ERPmin*, was necessary to identify as persistent behavior. *ERPmin* was defined as the shortest pacing interval during reference recordings using S1S2S3S4 stimulation where available. Otherwise, *ERPmin* was defined manually based on experience with the relevant species and disease state. If the majority of pAT points do not show repetition, impulse propagation events were assumed to self-terminate without re-excitation and the wave front was classified as non-repetitive. It should be noted that classification at this stage does not distinguish classical reentry from macroreentry, for example. Similarly, there is no inference of the source of the identified non-repetitive activity, which may have single or multiple origins, or be driven by sinus rhythm/pacing/automaticity/etc., A detailed description in the form of pseudocode for the implementation of the reentry classification procedure can be found in the Supplementary Material.

##### 2.8.3.4 Identifying origins of activation

Origins of activation presenting as sites of breakthrough on the imaged surface were evaluated throughout the full duration of each wave front label. Provisionally, pAT origins were found by assessing the spatio-temporal distributions of pAT minima (Supplementary Figure S3 for an example). Local pAT minima were determined as sites absent of preceding pAT points during an interval of *ERPmin* within the local 8 point neighborhood. However, considering pAT minima within a local neighborhood did not discriminate between multiple equivalent pAT points with a common origin such as when broad regions of tissue are activated simultaneously. These manifest either as a true breakthrough event or as false-positives due to continuity of propagation from a source peripheral to the region. Therefore false positive pAT origins were assessed using a connected components analysis to spatially group pAT minima points. Any of the points from the same pAT cluster with preceding pATs in the ERPmin interval and across an 8-point neighborhood would identify the whole pAT cluster as a false positive breakthrough sites.

Maps counting the occurrence of origins with dimensions [X,Y] were generated for each wave front such that *Origin*_*x,y*_ = *Origin*_*x,y*_ + 1. Complementary maps of the same dimensions were generated to show the location of the breakthrough center. The centeral pixel of origin pAT clusters were identified by averaging all horizontal and all vertical components of pAT point coordinates in each cluster to define mean focal origins.

##### 2.8.3.5 Dynamically probing complex activation and repolarization sequences

Thus far, we have shown how to compute and visualize activation and repolarization sequences for individual labels. However, we should consider that activation or repolarization events can occur simultaneously for different wave fronts located in distinct regions. Moreover, we only map the earliest pAT points for each wave front, ignoring re-excitation in repetitive wave front labels. Therefore, a single pAT map per wave front does not allow understanding of the interactions between wave fronts or transitions to persistent reentrant behavior. Moreover, a single AT map is not compatible with reentrant activity, which inherently has strong overlap of the activation sequence between cycles, particularly when wave fronts are out of phase and discordant. Thus, a dynamic method was developed to interrogate and visualize the ensemble spatial components in a stack of maps, each covering sub-windows of time for activation or repolarization sequences. Firstly, an image stack of dynamic maps with dimensions [X,Y] were generated based on the following steps: The first map was constructed by mapping the earliest time points (using the desired pAT or RT matrix). From the earliest time point, advancing in time through the matrix, the rest of the map was filled with time points until the first instance where the map already contained a time point. A new map was initialized firstly by populating the map with all values from the previous map. Then the time of the next matrix time points were found and replaced the values in the corresponding pixel of the new map. A new map was again initialized with the previous map values and the same procedure repeated until the full time window of interest of the time point matrix was mapped.

##### 2.8.3.6 Dynamic classification of activation sources of the leading wave front

Thus far, wave front labels have been broadly classified by their repetitive or non-repetitive sequences. But this is insufficient to understand the mechanisms underlying the arrhythmic event, such as myocardial reentry, macroreentry or short-coupled fast focal behavior, for example. Moreover, tachyarrhythmia are often unstable and evolving. Therefore a second detailed classification scheme is proposed to assess spatially and temporally the underlying source of activation for each pAT. In other words, which conduction source drove local propagation? Potential sources of conduction included: Passive activation from outside of the field of view; a breakthrough site on the imaged surface; a myocardial reentrant source; sources emanating from sites of discontinuous (tortuous) propagation and sources emanating from a wave front boundary.

**FIGURE 5:**
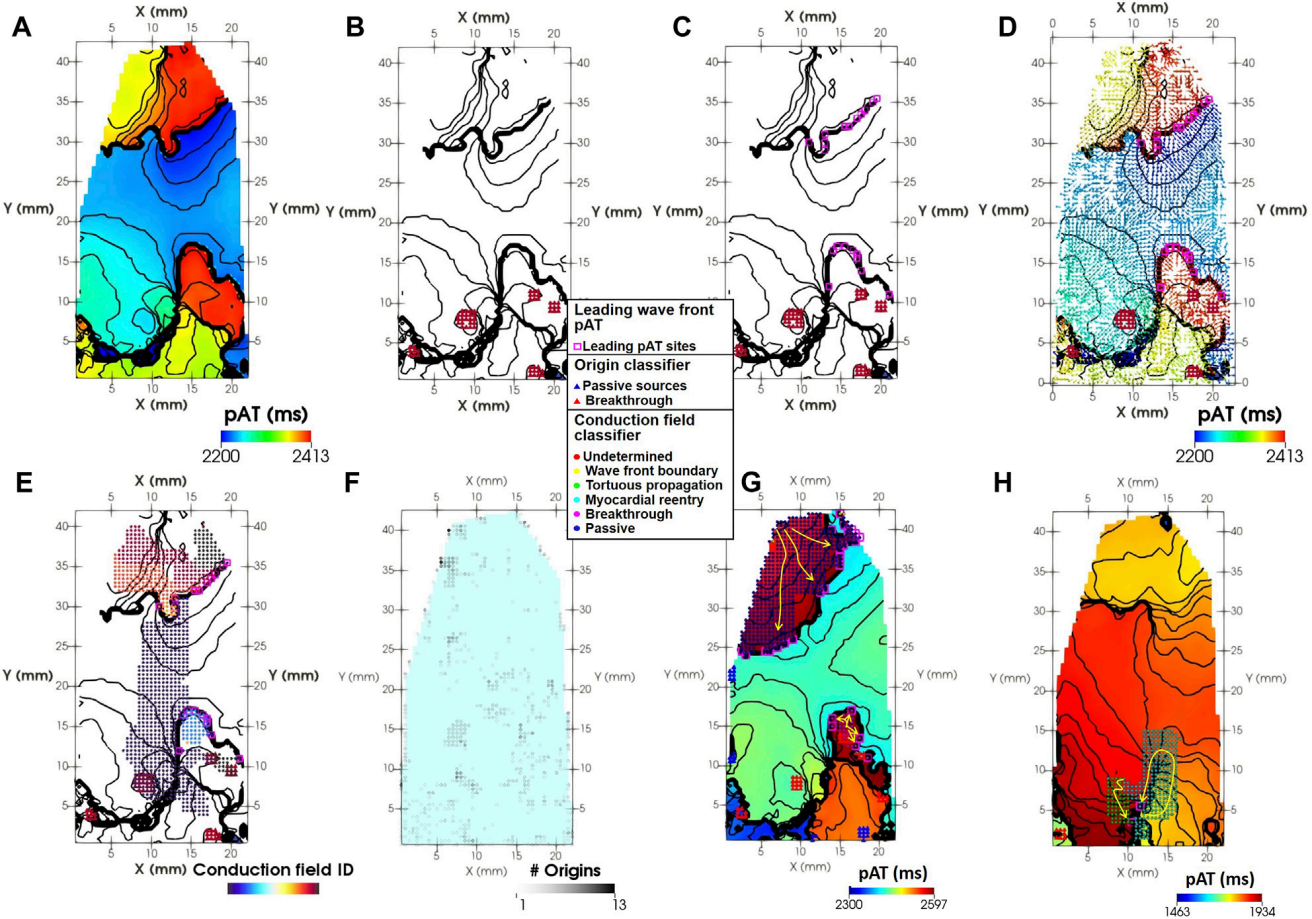
Spatial classification of activation sources. **(A)** A dynamic pAT map (left panel). **(B)** Origins of activation for breakthrough (red triangles) and passive (blue triangles) sources. **(C)** Leading pAT points of the active wave front (pink squares). **(D)** Conduction vectors oriented in the direction of local propagation overlaid on **(C)**. **(E)** The conduction fields of leading pAT points. **(F)** Number of accumulated breakthrough or passive origins throughout wave front #4. **(G)** A pAT map shows leading wave front pAT sites (purple squares) with conduction fields emanating from a single breakthrough (red triangles) and passively from outside of the imaged field of view. **(H)** The leading wave front during tachyarrhythmia was also maintained by reentrant propagation through myocardial pathways or regions of tortuous propagation. Yellow arrows indicate the local direction of propagation.

Each pAT was individually classified by activation source in dynamic pAT maps (Figure 5A). Prior to classification, we identified if origins of activation (see Section 2.8.3.4 Identifying origins of activation) reflect breakthrough sites or origins of passive activation located at the periphery of the masked region of interest (Figure 5B). Pixels were selected for classification by identifying maximum pAT values along the leading wave front edges (Figure 5C). In addition, as shown in Figure 5D, local vectors of conduction normal to pAT contours were determined across the dynamic map using a finite different method (Cantwell et al., 2015). In order to identify the origin of each pAT value, the full surface area of tissue involved in the activation sequence (conduction field) leading directly to each maximum pAT site were identified as follows:

1) The first pAT from the leading wave front, found at (*x,y,t*) was annotated with a unique conduction field label.
2) Evaluate connectivity with adjacent unlabeled pAT points in a local 8-point spatial neighborhood. Neighboring pAT values within the time interval extending from t–*θ*_*t,CB*_ to t were considered. In the absence of eligible pAT neighbors, go directly to (3). Eligible pAT points were evaluated for conduction vectors intersecting *pAT* (*x,y,t*). Unlabelled intersecting pAT pixels were assigned the same conduction field label value as *pAT* (*x,y,t*) and the neighboring pAT point coordinates for matrix *X*,*Y*,*T* were added as the first elements in a queue; then go to (3).
3) Remove the last pAT coordinates from the queue and repeat (2) until there are no more elements in the queue. Repeat from (1) for the next leading wave front pAT value.

A classification scheme determined the origins of each identified conduction field driving the leading wave front pAT sites (Figure 5E). Evaluating each pAT point in conduction field, identify pAT points matching the following criteria for each class.

1) Breakthrough: One or more pAT points co-localize with one or more breakthrough activation origins (Figures 5F, G).
2) Passive: One or more pAT points co-localize with one or more passive activation origins (Figures 5F, G).
3) Myocardial reentry: Any number of conduction field pAT points with neighboring pAT points of the same conduction field with differences in pAT values > ERPmin (Figure 5H).
4) Tortuous propagation: One or more conduction field pAT points with neighbors within the time interval extending from t–*θ*_*t,CB*_ to t, but absent of intersecting conduction vectors (Figure 5H).
5) Wave front boundary: One or more conduction field pAT points absent of intersecting conduction field neighbors and neighboring pAT points from a different wave front label.

### 2.9 Statistical analyses

The performance and robustness of the signal processing pipeline was evaluated by varying the signal-to-noise ratio (SNR) of optical mapping recordings during tachyarrhythmia induction (*N* = 5, see Supplementary Figures S4–S8 for examples at baseline conditions). Non-parametric analyses of variance Friedman tests were used to identify statistical differences of signal characteristics and signal processing outputs across SNR populations. A multiple comparison assessment was also performed to compare results for individual SNR populations versus the baseline SNR population (established using a cut-off of 60Hz for a butterworth lowpass filter). Spatial correlations by pixel-to-pixel linear regression analyses were used for simulation data to compare eAT with uAT and pAT maps. Statistical differences of linear regression values between uAT and pAT performance was evaluated using the paired *t*-test. For all tests, statistical significance was determined when *p* < 0.05.

## 3 Results

### 3.1 AT mapping

**FIGURE 6:**
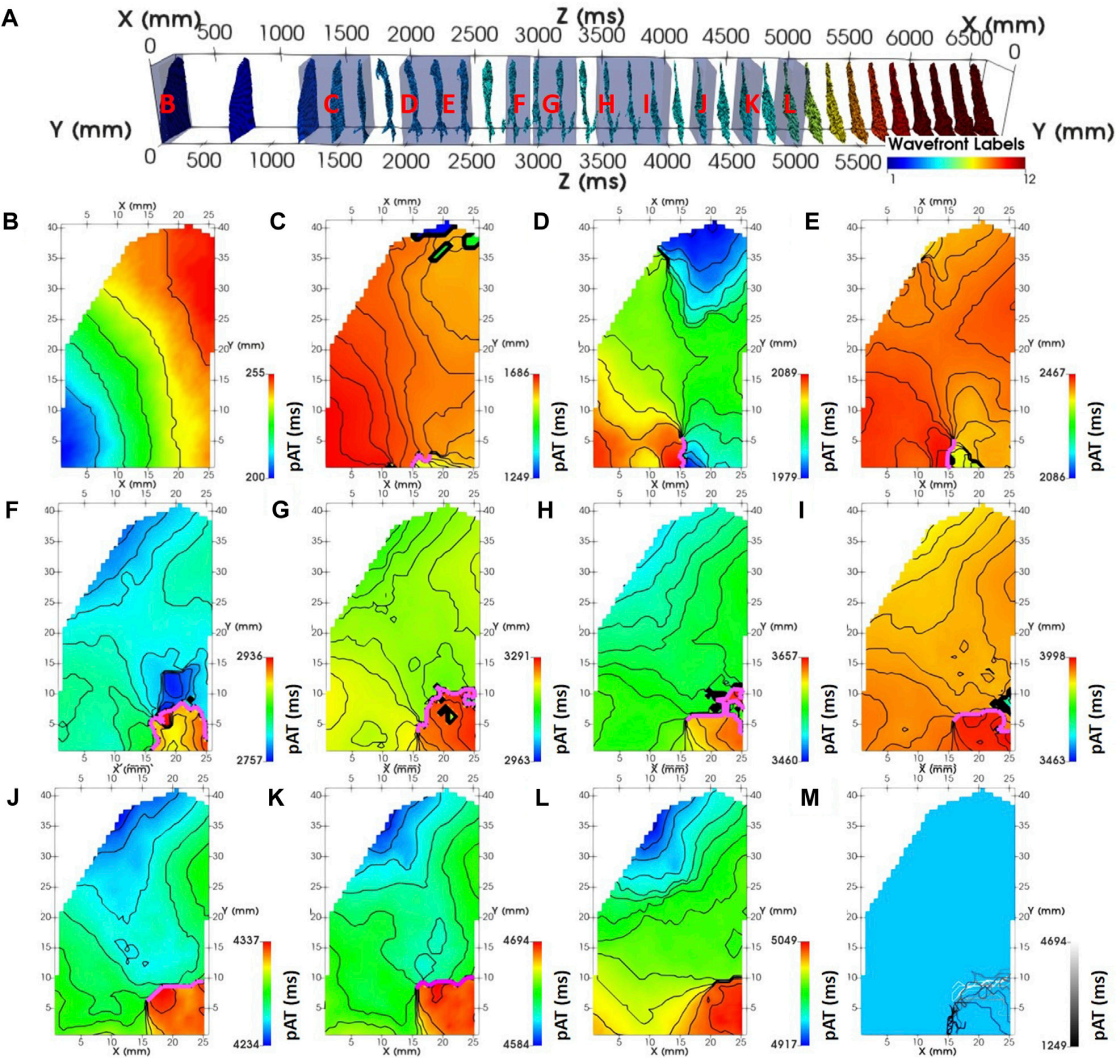
Dynamic mapping of experimental complex activity. **(A)** A spatio-temporal distribution of pAT points spanning pacing, induction and reentry phases of the experimental optical acquisition. pAT maps were dynamically probed for wave front labels (#): 1 **(B)**, 3 **(C)**, 5 **(D–F)**, 6 **(G–K)**, and 7 **(L)**. Lines of conduction slowing where the direction of maximal gradient exceedes 50 ms are annotated (pink lines). pAT windows are labelled in panel **(A)** (red). **(M)** Lines of conduction slowing superimposed. Lines are colored according to their average AT.

Figure 4B showed that the pAT matrix derived from the experimental example was composed of distinct wave front labels with the conduction block parameter set to 76 ms. The experimental example acquisition of tachyarrythmia analyzed was 6,700 ms. The analysed arrhythmic episode was 5,211 ms in length, 14 out of 28 beats were classified as repetitive activity. Mean cycle lengths were 186 ms. Image stacks of pAT maps incrementing through the pAT matrix from the experimental example recording enabled dynamic visualization of the full activation sequence (Supplementary Video S1). Figure 6 shows extracts of pAT maps from the image stack and the corresponding time windows (Figure 6A). Figure 6B shows the pAT map for wave font #1 during steady-state pacing prior to the induction of reentry. Total pAT across the region of interest was 56 ms and composed of a regular near-planar wave front that propagated unperturbed from the lower left to upper right of the pAT map. A sub-region of pAT points from wave front #4, approximately 200 ms after the shock were projected on to a pAT map (Figure 6C). In contrast to uAT mapping (Figure 1F) pAT found a preferential activation of the lower left region of the map, coinciding with the region of early activation during basal pacing. The wave front subsequently propagated in a counter clockwise movement with a maximal local pAT gradient of 75 ms/mm observed between neighboring pixels and where pAT contours converged to a single site at the core of the circular movement. Over 20 arrhythmic cycle lengths, every second cycle of the arrhythmic episode was plotted as pAT maps (Figures 6D–6L), until a distinct near-planar wave front was observed (Figure 6L). The AT sequence was consistently characterized by a counter clockwise rotation. It was observed that each reentrant cycle was accompanied by a region of local pAT gradients >50 ms/mm, indicating conduction slowing. The locally enhanced pAT gradient was primarily vertically orientated at the bottom of the pAT map during wave front #4 (Figures 6C–E), but shifted towards the right border of the pAT map coincided with the transition to wave front #5 (Figure 6M).

### 3.2 Origins of activation

In an additional experimental preparation, the ventricular wedge from sheep was paced at a frequency of 6 Hz (Supplementary Figure S3). pAT minima were identified for each wave front label (Supplementary Figure S3). The activation sequence globally propagated from a single region of the myocardial surface from left to right (Supplementary Figure S3B). Yet the earliest pAT region was shared between two sites (Supplementary Figure S3C). These coincided with two electrodes used for bipolar stimulation. However, two sites of origin distal to the stimulation location were also observed. Initial computation revealed pAT origin clusters, indicating simultaneous activation of areas exceeding a single pixel (Supplementary Figure S3D). Supplementary Figure S2E shows the pAT origin clusters reduced to the estimated center-most pixel, representing a local single pAT origin site.

### 3.3 Mapping repolarization properties

Supplementary Figure S4A shows pAT values derived from recordings of multiple short-coupled stimuli and ectopic activity at the onset of self-sustained tachyarrhythmia following S1S2S3S4 pacing (see Section 2.5 Pacing protocols). This pacing regime is composed of irregular coupling intervals that gave way to highly varying total pATs ranging from 42 ms (S1) to 334 ms (ectopic beat A). Despite the irregular coupling intervals and total pAT exceeding the shortest coupling interval (S3-S4, 215 ms), RTs could be determined across the imaged field of view (Supplementary Figure S4B). A user-defined window of 80–400 ms following pAT was used to refine repolarization time estimates. Resulting RT gradients (maximum–minimum) ranged from 72 ms (S1) to 424 ms (ectopic beat A). Pixels assigned both pAT and RT values were subsequently used to derive APD (Supplementary Figure S4C).

### 3.4 Wave front classification

**FIGURE 7:**
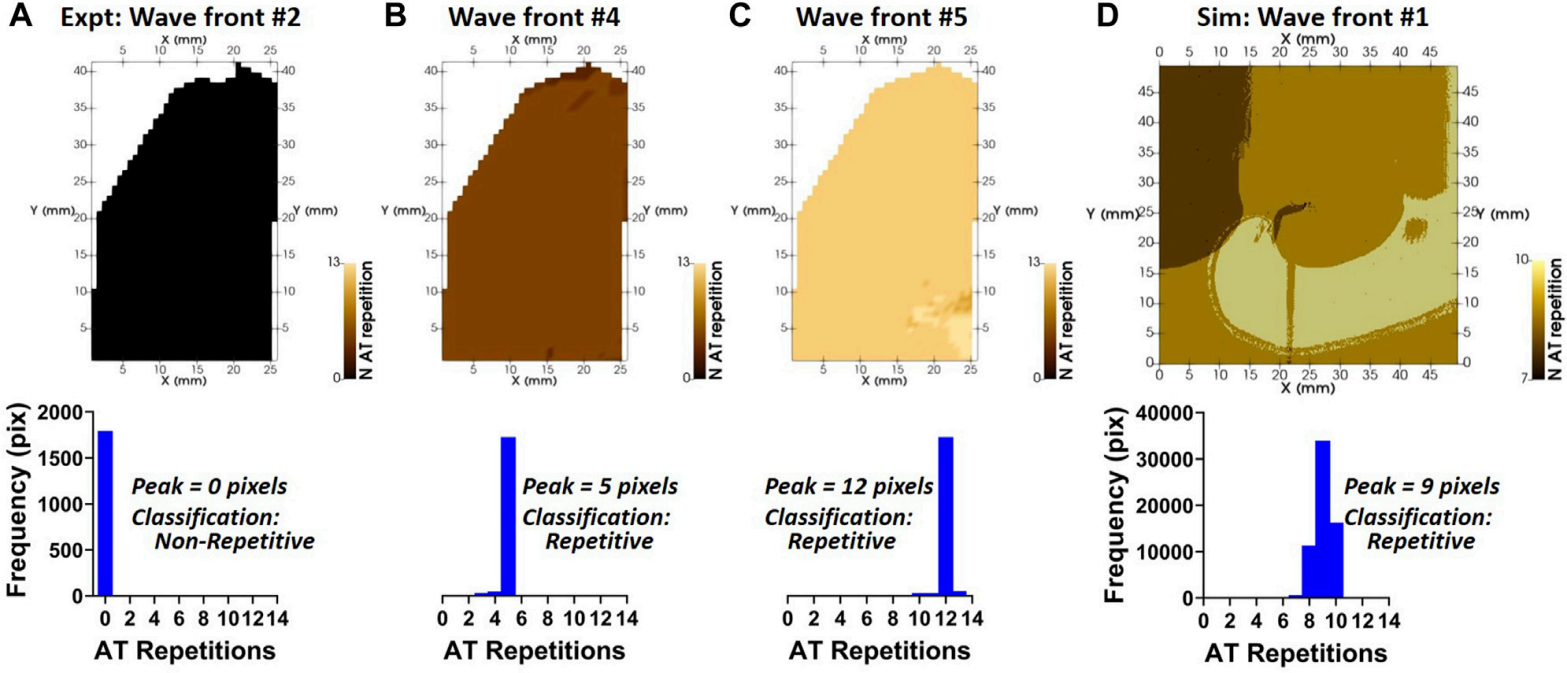
Repetitive/non-repetitive classification of wave fronts. **(A—D)** Wave fronts shown in Figure 5 were classified as repetitive or non-repetitive. The spatial distribution of the number of repetitions of activation for each wave front (upper panels) and their histograms (lower panels) are shown. Labels were classified as repetitive if the histogram peak of activation repetitions was ≥1.

Labelled wave fronts derived from the experimental and simulated acquisitions underwent classification in to repetitive and non-repetitive activation sequences. Figure 7A shows a map and histogram of pAT repetitiveness for wave front #2 of the example experimental recording under basal stimulation. Zero pixels observed repetitive pAT points with intervals exceeding a minimum ERP parameter, which was set to 200 ms. In this case, we did not have recordings of short-coupled stimulation intervals to identify the local ERP. Therefore, a minimum ERP parameter was estimated to equate 80% of the minimum action potential duration (250 ms) during basal stimulation. Wave front #2, was consequently classified as a non-repetitive activation sequence. Wave front #4, which was initiated by the cross-field shock and the activation sequence was maintained for a period of 1,018 ms. Wave front #4 was found to have almost uniform repetitiveness of 5 pAT repetitions across all pixels (Figure 7B). A dominance of pAT repetition across pixels underlay the classification of wave front #4 as repetitive. Similarly, wave front #5 was classified as repetitive with a predominance of 12 repeated pAT points during a total activation period of 2,297 ms (Figure 7C). Concordantly, a computer model of repetitive behavior was accurately classified as repetitive through computation of the dominant pAT repetitiveness factor (Figure 7D). Local classification of the origin driver provided insight in to the spatial organization of arrhythmia and the temporal evolution underlying transitions between activation sequences, notably from wave front #4 to #5.

In the experimental example, origins of activation were detected following the onset of the tachyarrhythmic episode (wave front #4) both as breakthrough sites and at the periphery of the imaged tissue (Figure 5A). In part, breakthrough sites contributed to the progression of the wave front in the lower portion of the pAT map as observed by overlapping conduction fields with breakthrough sites (Figure 5B). The upper portion of the pAT map was primarily driven by passive impulse propagation emanating from outside of the imaged tissue in the upper left corner. Figure 5C shows that the wave front was also driven by myocardial reentry and tortuous propagation in the regions of slowest conduction. Throughout the full arrhythmic episode, there was a predominance of passively activated wave front (74.1%). This was primarily attributed to propagation extending from the top of the mapped area throughout the recorded arrhythmia episode. However, 22.3% was attributed to breakthrough sites of origin, observed as an important mechanism for maintenance of re-excitation of the lower portion of the map during wave front #4 and #5. To a lesser extent, the tissue was maintained through myocardial reentrant pathways and sites of tortuous propagation.

### 3.5 Robustness of phase window-derived AT mapping

**FIGURE 8:**
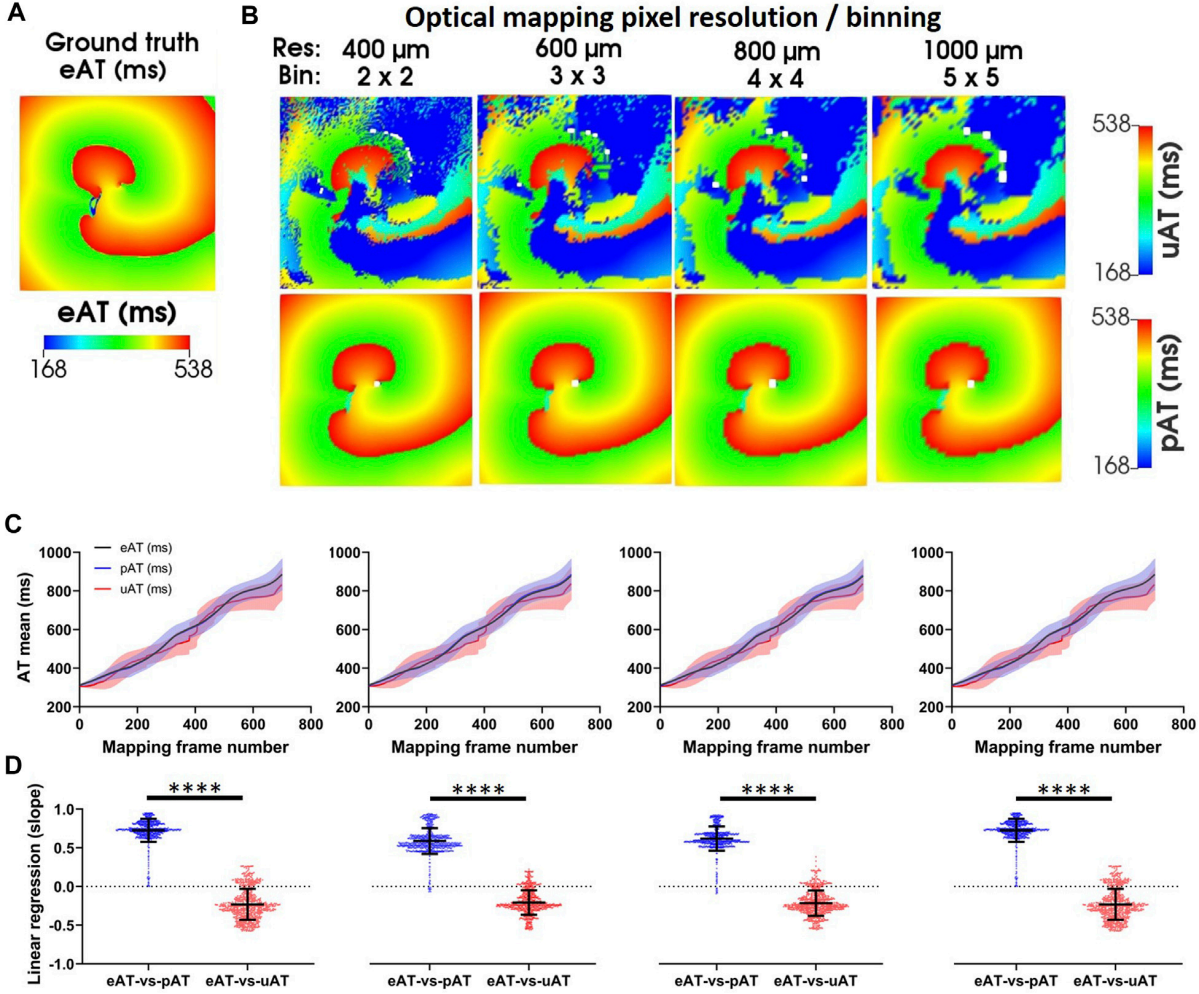
Simulations of pAT mapping and image resolution. **(A)** Ground truth eAT derived from a simulation of the underlying electrical action potential. **(B)** Optical mapping signals were derived from electrical simulations in **(A)**. The effects of image resolution evaluated on uAT and pAT derived from optical mapping signals. Optical mapping signals were derived from downsampled electrical simulations to simulate reduction of image resolution from 400 μm to 1,000 µm. **(C)** Mean ± standard deviation eAT, uAT, and pAT from dynamic AT maps of the full simulation. **(D)** Linear regression of pixel-to-pixel correlations of eAT with uAT and pAT.

The core methodology of this processing and analytical pipeline centers on the capacity of pAT mapping to reliably and reproducibly detect AT points in complex dynamic electrical behavior. For comparison to experimental measurements, a repetitive activation sequence was induced in a computational model. A pAT matrix was constructed over the duration of the simulated time window of 2 s (Figure 4C). The activation sequence was established to be a single wave front (#1), indicating a self-sustained arrhythmia. Figure 8A shows an activation map of the underlying electrical action potential (eAT), representing the ground truth activation sequence. A clockwise rotating activation sequence was observed on the eAT map. Figure 8B shows equivalent maps for uAT and pAT at varying image resolutions. Maps of uAT shows a complex and fragmented activation sequence with a total AT (95%–5% AT) of 362 ms, compared to 185 ms for eAT. However, pAT much more closely resembled the spiral activation sequence of the ground truth. Total AT for pAT was 192 ms. Sensitivity of the signal processing pipeline was tested on a series of simulations following down sampling of the ground truth electrical simulation and generation of optical signals with reduced resolution from 400 μm to 1,000 µm. Both uAT and pAT was compared across the down sampled simulated data sets, but there was no impact on the overall activation sequences detected using mapping methods (Figure 8C) and linear regression analysis comparing eAT with uAT and pAT showed consistently significantly higher correlations with eAT than uAT (Figure 8D).

**FIGURE 9:**
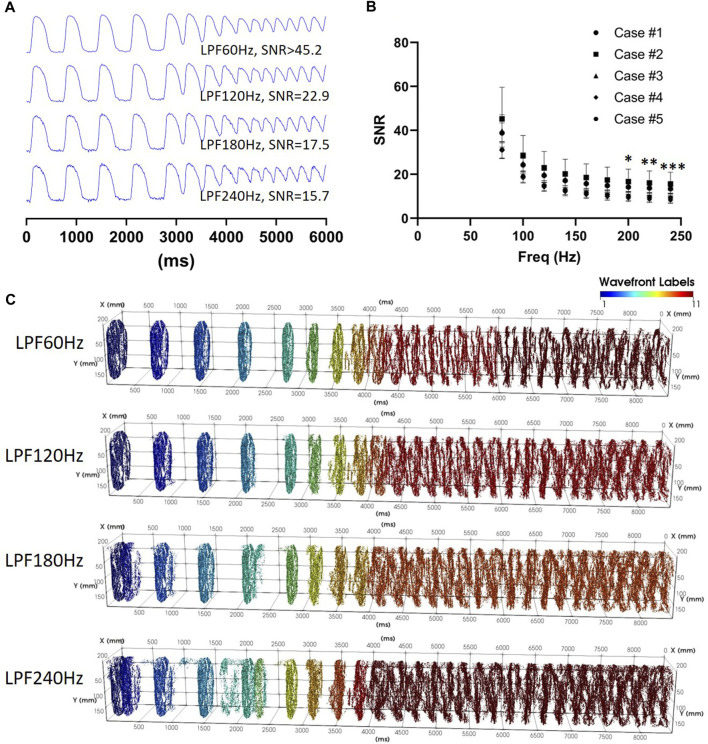
Influences of the signal-to-noise ratio. **(A)** Action potential traces with varying lowpass filter cut-off frequencies. **(B)** Mean ± standard deviation signal-to-noise ratios for experimental optical mapping recordings during S1S2S3S4 stimulation and tachyarrhythmia induction (N = 5). **(C)** Spatio-temporal distribution of pAT points corresponding to recordings used in A.

Our signal processing framework relies on robust automated determination of *θ*_*t,CB*_ using a reference recording during S1S2S3S4 stimulation, the separation of pAT points in to coherent wave front labels and the capacity to reliably classify repetitive wave front activity. Table 1 summarizes the experimental parameters for implementation in five cases from left ventricles of sheep. The shortest stimulation interval ranged from 190–260 ms across all cases. Despite this variation, the conduction block parameter remained consistent with a mean (±standard deviation) of 59.2 ± 17.0 ms. Supplementary Figures S5–S9 showed pAT distributions, wave front labelling and pAT maps of each case during the transition from the end of a train of S1 pulses to short-coupled stimulation and to post-stimulation events. Despite broad total pAT events overlapping with stimulation intervals, activation sequences elicited by each stimulation pulse were successfully isolated and grouped in to individual wave fronts for each experimental case. Table 1 shows that signal-to-noise ratios of recordings were progressively reduced from >37.2 to 12.1 to evaluate pAT-sensitivity to noise (Figures 9A, B). Lowering signal-to-noise ratios reduced signal regularity indices significantly when limiting signal filtering to low pass filter cut-off 180 Hz. Concordantly, phase-derive AT window estimation also observed marginal reduction of regularity indices. Despite reduced quality of signals and signal windowing, output parameters were only significantly influenced by noise amplification using lowpass filter cut-offs >180Hz. Decreases in the estimations of *θ*_*t,CB*_ and augmented θ”_*dF*⁄*dt*_ were observed. Similarly, the number of wave fronts detected, pAT points and breakthrough sites were preserved using filtering cut-off <180Hz (Figure 9C).

**TABLE 1:** Repeated use and robustness of the novel pAT mapping framework in experimental cases. Ventricular arrhythmia was induced in five experiments using an S1S2S34 induction protocol.

Lpf cut-off frequency (Hz)	60	80	100	120	140	160	180	200	220	240	*p*-Value
Stimulation protocol											
Minimum interval of short-coupled S1S2S3S4 stimuli (ms)	224 ± 23.4	224 ± 23.4	224 ± 23.4	224 ± 23.4	224 ± 23.4	224 ± 23.4	224 ± 23.4	224 ± 23.4	224 ± 23.4	224 ± 23.4	NS
Mean signal characteristics											
SNR	>37.2	37.2 ± 6.1	23.1 ± 4.2	18.4 ± 3.7	16.0 ± 3.4	14.6 ± 3.3	13.7 ± 3.2	13.0 ± 3.2*	12.5 ± 3.1**	12.1 ± 3.1***	<0.0001
AP signal Dominant frequency (Hz)	1.7 ± 0.4	1.7 ± 0.4	1.7 ± 0.4	1.7 ± 0.4	1.7 ± 0.4	1.7 ± 0.4	1.7 ± 0.4	1.7 ± 0.4	1.7 ± 0.4	1.7 ± 0.4	NS
AP signal regularity index	0.62 ± 0.09	0.60 ± 0.09	0.60 ± 0.09	0.59 ± 0.09	0.59 ± 0.09	0.59 ± 0.09	0.59 ± 0.09*	0.58 ± 0.09**	0.58 ± 0.09***	0.58 ± 0.09****	<0.0001
pAT characteristics											
pAT window dominant frequency (Hz)	2.2 ± 1.2	2.2 ± 1.2	2.2 ± 1.2	2.2 ± 1.2	2.2 ± 1.2	2.2 ± 1.2	2.2 ± 1.2	2.2 ± 1.2	2.2 ± 1.2	2.2 ± 1.2	NS
pAT window regularity index	0.37 ± 0.12	0.35 ± 0.12	0.35 ± 0.11	0.33 ± 0.12	0.33 ± 0.12	0.32 ± 0.12	0.32 ± 0.12*	0.31 ± 0.12**	0.33 ± 0.16*	0.33 ± 0.15**	0.0004
θ”_*dF*⁄*dt*_	0.00039 ± 0.00013	0.00045 ± 0.00015	0.00049 ± 0.00016	0.00053 ± 0.00018	0.00057 ± 0.00019	0.00060 ± 0.00020	0.00062 ± 0.00021*	0.00064 ± 0.00023**	0.00066 ± 0.00024***	0.00068 ± 0.00024****	<0.0001
*θ*_*t,CB*_ (ms)	59.2 ± 17.0	56.2 ± 14.8	53.6 ± 13.7	54.0 ± 13.9	51.6 ± 12.6	52 ± 12.7	49.6 ± 12.3*	48.8 ± 12.1*	48.4 ± 11.6*	44.4 ± 12.3*	0.0002
N wave fronts	19.8 ± 15.0	17.8 ± 15.3	15.4 ± 13.2	14.2 ± 12.9	15.2 ± 13.9	16.4 ± 13.2	11.2 ± 2.7	13.8 ± 13.2*	16.0 ± 12.9	15.6 ± 13.6	0.0156
N AT points	216,810 ± 179,447	223,596 ± 174,066	230,518 ± 167,108	239,869 ± 160,573	248,019 ± 157,367	257,069 ± 155,073	273,621 ± 152,972	275,621 ± 150,720	284,561 ± 151,429	291,159 ± 151,319**	0.0015
N breakthrough sites	2,461 ± 1899	2,580 ± 1839	2,601 ± 1719	2,657 ± 1701	2,733 ± 1,685	2,758 ± 1,698	2,740 ± 1858	2,980 ± 1901	3,112 ± 1846**	3,222 ± 1832***	0.0004
Wave front repetitiveness											
N Non-sustained wave fronts	17.2 ± 15.7	15.2 ± 16.3	12.6 ± 13.8	12.0 ± 12.6	12.2 ± 13.6	13.8 ± 14.3	8.8 ± 3.3	12.0 ± 13.1	13.6 ± 13.3	13.8 ± 14.4	NS
N sustained wave fronts	3.6 ± 1.5	4.2 ± 1.3	3.6 ± 1.5	4.2 ± 1.9	4.2 ± 1; 3	4.2 ± 1.3	3.2 ± 1.3	3.3 ± 1.2	3.4 ± 1.5	4.4 ± 1.3	NS
Classification of the origins of the local wave front											
Breakthrough (%)	9.7 ± 1.3	10.6 ± 1.4	10.7 ± 1.1	11.2 ± 1.9	10.4 ± 0.8	10.3 ± 0.6	9.9 ± 0.6	10.1 ± 0.5	10.1 ± 0.6	10.2 ± 0.7	NS
Passive (%)	4.3 ± 1.3	4.5 ± 1.4	4.6 ± 1.3	4.8 ± 1.3	4.6 ± 1.1	4.5 ± 1.1	4.5 ± 1.1	4.6 ± 1.2	4.5 ± 1.0	4.5 ± 0.9	NS
Myocardial reentry (%)	0.5 ± 0.5	1.0 ± 1.2	1.5 ± 1.8	1.9 ± 2.3	2.1 ± 2.6	2.4 ± 3.0	2.6 ± 3.3*	3.1 ± 3.8**	3.1 ± 4.0*	3.6 ± 4.0**	0.0001
Tortuous propagation (%)	85.5 ± 2.7	83.8 ± 3.4	83.2 ± 3.5	82.0 ± 4.1	82.9 ± 4.1	82.9 ± 4.1	82.9 ± 4.4	82.2 ± 4.7	82.3 ± 4.4*	81.6 ± 4.2**	0.0157
Wave front boundary (%)	0.018 ± 0.026		0.008 ± 0.012	0.038 ± 0.076	0.008 ± 0.011	0.015 ± 0.018	0.023 ± 0.024	0.02 ± 0.021	0.019 ± 0.027	0.0273 ± 0.035	NS

Data are mean ± standard deviation and *N* = 5 for all cases. Statistical significance was considered if *p* < 0.05. *p < 0.0332, **p < 0.0021, ***p < 0.0002, ****p < 0.0001.

### 3.6 pAT mapping of tachyarrhythmia in chronic myocardial infarction

**FIGURE 10:**
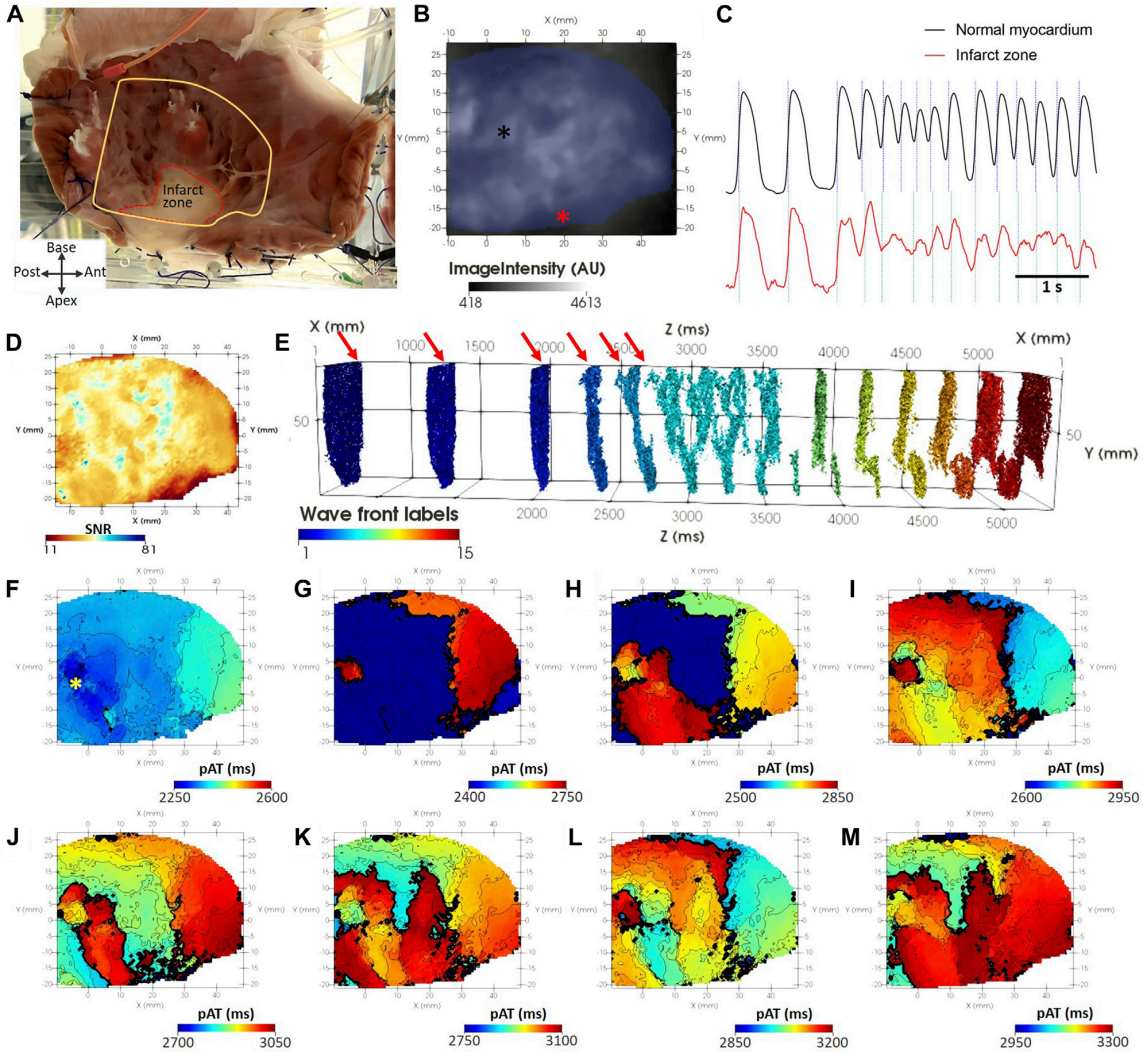
pAT mapping tachyarrhythmia in chronic myocardial infarction. **(A)** The endocardial surface was subject to optical mapping, encompassing a region of scar in the ventricular apex. **(B)** Background optical mapping signals. **(C)** Optical mapping traces taken from normal myocardium [black asterisk in **(B)**] and the scar region [red asterisk in **(B)**]. **(D)** Map of SNR from the same optical mapping recording. **(E)** pAT matrix with labelled wave fronts and indicating the timing stimulation pulses (red arrows). **(F–M)** Progressive pAT maps during the onset of tachyarrhythmia.

The endocardial surface of the structurally remodeled was imaged by optical mapping (Figures 10A, B). Figure 10C showed that optical mapping signals were observed within the infarct zone indicating surviving functional myocardium and conduction within the structurally remodeled region, although SNR was reduced (Figure 10D). Nevertheless, a pAT matrix was derived. *θ*_*t,CB*_ was automatically detected as 62 ms and *ERPmin* was 180 ms. These parameters enabled coherent wave front allocation throughout the mapped surface during S1S2S3S4 stimulation and tachyarrhythmia onset (Figure 10E). Planar wave front propagation from the posterior left ventricular free wall induced by S2 (Figure 10F) was followed by passive activation from the anterobasal region (Figure 10G). Simultaneously, impulse propagation from S3 stimulation collided with the passive wave at the infarct zone (Figure 10H). This was followed by apex-to-base propagation in to the posterolateral base (Figure 10I). The excitatory wave front continued to advance to the anterior base and anterior apex (Figure 10J), colliding with an S4-driven impulse. From the late activated apical scar region, the wave front emanated towards the base along a narrow trajectory with an antero-lateral aspect (Figure 10K). The wave front successfully propagated to the base where it diverged towards the posterior and anterior left ventricle (Figure 10L). Figure 10M showed both wave fonts circumventing the lateral left ventricle to again converge at the apical scar region in a figure of eight formation. Interestingly, pAT mapping of the scar tissue region was incomplete in Figure 10H requiring almost 200 ms to maximally activate (Figure 10I). The pAT map could distinguish individual pathways of fast and slow conduction within the structurally remodeled tissue.

## 4 Discussion

The framework for a novel analytical approach has been developed for AT mapping of complex electrical behavior recorded using optical mapping. A first critical aspect was to accurately probe activation events. For each image pixel, a Hilbert Transform phase analysis revealed the time intervals of action potential upstrokes. In turn, pAT events were elucidated for each upstroke based on signal morphology and slope profile. The spatio-temporal distribution of pAT events were assigned groups corresponding to individual wave fronts. This enabled individualized analysis of the conduction properties of wave fronts. The corresponding time of repolarization and spatial distributions were also derived. Moreover, each wave front could be classified in to probable repetitive or non-repetitive propagating fronts. An approach for dynamically viewing the ensemble activation sequence throughout complex events was developed. This aided detailed interrogation of wave front interactions and classification of the wave front progression based on the underlying source of impulse propagation, irrespective of the complexity and irregularity of electrical organization. In addition, the pAT algorithm was thoroughly evaluated for sensitivity to SNR and image resolution. Finally, the algorithm was further tested on an experimental model of chronic myocardial infarction in sheep.

The aim of our pAT-based analysis of complex optical mapping signals is 2-fold: i) To accurately deduce the propagation sequence while conserving a high spatial resolution and ii) To thereby inform on the critical properties of arrhythmia and functional pro-arrhythmic factors. pAT maps can report on the source of activation for any given pAT point (Figures 5, 8). Moreover, pacing modalities are important tools for manipulating the AT or RT sequences to characterize functional substrates, induce arrhythmia or entrain and arrest arrhythmic behavior. Generally, high pacing frequencies or variable stimulation intervals are applied, which incurs further complexity to interpret and isolate the relevant AT events. Generally, AT mapping relies on adequately selecting a time window of interest, identification of a suitable time reference and robust interpretation of the signal morphology. Depending on the approach selected to probe ATs, conflicting information can often times be produced (Walton et al., 2012; Tomek et al., 2021). This effect can be further confounded when signal complexity increases (Bishop et al., 2007; Asfour et al., 2011; O’shea et al., 2019). Therefore, identifying a robust approach to appropriately identify the window of activation and accurately interpret AT irrespective of the signal complexity underlay signal processing automation without prior understanding of the signal morphology.

Enhancing the versatility of our processing pipeline, this framework provided automatic determination of some critical properties of optical signals, such as maximal pAT gradients that determine thresholds of conduction block and tissue refractoriness. Moreover, automated assignment of such parameters can be derived on an individual basis for optical mapping images. This will enable the analytical procedure to adapt dynamically to changing conditions, such as rhythm or effects of ion channel-targeting drug treatments. Furthermore, the approach for determination of pAT was developed to maximally retain propagating wave front information by evaluating the upstroke morphology and signal derivative (Figure 3). The maximum derivative of the action potential upstroke is known to precisely reflect AT on the imaged surface of tissue (Walton et al., 2012). Moreover, the orientation of the wave front relative to the imaged surface can be inferred from the normalized amplitude of the maximal derivative on the upstroke, termed *V*_*F*_ * (Zemlin et al., 2008). *V*_*F*_ * is a byproduct of the derivation of pAT in our processing pipeline, which can therefore be exploited when implementing this framework. Biphasic upstroke morphologies have been shown to correspond to propagation through distinct myocardial pathways found within the tissue volume contributing to the same optical signal (Fedorov et al., 2010). Secondary pAT components likely inform on remnant late propagating electrical impulses (Kertes et al., 1984). Clinically, late potentials with slow conduction are thought to underlie numerous electrical disorders (Haïssaguerre et al., 2019a; Haïssaguerre et al., 2019b). In this context, information-loss through signal reduction to a single AT event for any given upstroke would likely exclude the critical arrhythmic pathway in favor of the principal propagating wave front.

Optical mapping has seen substantial development over recent years, particularly in the event of near-infrared voltage-sensitive dyes (Matiukas et al., 2007). Using near-infrared excitation wavelengths incur several differences to more conventional blue/green excitation optical mapping. Firstly, longer wavelength light better penetrates biological tissue as a result of reduced absorption and scattering properties. This can more easily result in substantial transillumination light if a photodetector (CCD/CMOS camera, for example) was simultaneously imaging the opposite surface to the one being illuminated (Baxter et al., 2001). Moreover, reduced attenuation and scatter increases the overall optical integration volume contributing to fluorescence. This means that a larger sub-surface volume of tissue contributes to the optical signal in epi-fluorescence mode and to an even greater extent when imaging transilluminated light. This further contributes to blurring of the optical action potential upstroke. Despite this, we showed in (Walton et al., 2012) that AT, defined as the maximal derivative of the optical action potential upstroke effectively approximates the true electrical AT irrespective of the excitation light wavelength in the range of 530 nm (green) to 660 nm (near-infrared). To our knowledge, there are no known alternatives of the fundamental approach to define AT that further improves this estimation. This strongly supports the versatility of the framework that we propose and its compatibility with near-infrared imaging, as well as transilluminated signals. We further demonstrated that image resolution has no impact on pAT computation and that the proposed pipeline is relatively insensitive to signal noise; an important factor that often depends on the experimental conditions and choices of voltage-sensitive dyes used.

Arrhythmia events can occur suddenly or as a result of gradual adaptation over several heart beats. Therefore, to prevent further information loss our pipeline avoids other forms of signal reduction such as ensemble averaging of action potentials. Yet, analysis of each propagation event may be complicated by interference from adjacent and spatially overlapping wave fronts and repolarization times. That being said, the interactions of independent wave fronts and particularly the influence of repolarization heterogeneity and refractoriness on subsequent electrical responses is crucial to determine how arrhythmia is maintained. Therefore, in order to optimize the diagnostic yield when mapping the arrhythmia mechanism, this framework probes the pAT matrix (and subsequently derived RT matrix) both at the individual wave front level and through a customized dynamic window of the ensemble time-based data fields. This allowed substantial mechanistic insight in to the causal activation sequence and origin of the leading wave front at the individual pixel level. Probing the conduction field of aberrant wave fronts provides deep understanding of the regions of tissue involved and the source of the activity. This insight will aid to identify arrhythmia organization and improve investigations of optimal therapeutic interventions on arrhythmia sources.

We have presented our own analytical tools to evaluate and classify arrhythmic behavior within this framework. However, our tools also serve to extract detailed activation sequence data pertinent to existing analytical approaches. Isolating wave fronts is a necessary post processing step for the multiplicity metric (Rogers et al., 1997a; 1997b). Similarly, the reentry vulnerability index depends upon activation and repolarization times to estimate local sites susceptible to re-excitation. In our framework, wave front classification of the repetitiveness of activity rapidly provides insight of the life-time of persistent versus non-persistent activity, which may be used analogously with rotor life-time analysis (Kay et al., 2006). Figure 6 showed that local points of rotation of the activation sequence were associated with large pAT gradients (>50 ms/mm). Moreover, conduction field analysis of wave front origins did not rely on the gradient point or line and was therefore not hindered by complex gradient organization or discontinuity that can hamper phase singularity detection (Arno et al., 2021). Therefore, our framework provides a versatile unified solution to analyze spatio-temporal dynamics of cardiac arrhythmias.

### 4.1 Limitations

The current framework has been parametrized to analyze optical mapping signals derived from voltage-sensitive dyes, accommodating action potential signal morphologies. Yet, the foundational approach to window individual activation events based on phase responses can likely be applied more diversely across dynamic imaging and recording modalities. More so, this proof-of-principle study focused on data obtained only from sheep ventricles. However, each procedure of this pipeline was designed on the premise of being applicable to the broader spectrum of species or anatomical regions (e.g., atria vs. ventricles) and therefore action potential morphologies. The capacity to adapt from the basal action potential to complex and non-periodic fibrillatory activity within the same recording, insensitivity to image resolution, noise-handling and application in pathological settings is a strong indicator of the framework’s versatility and robustness.

Our analytical framework proposes a novel approach to estimate the threshold of conduction block, *θ*_*t,CB*_, although a single threshold value generalizes the conduction limitations across the imaged field of view. However, the maximal electrical impulse transmission delay across structural substrates is likely heterogeneous. Cases in this study showed that regions of high pAT gradients represents only a small subset of the total activation sequence (∼5%, Supplementary Figure S3). The challenge is to identify the excitable gap corresponding to vulnerable sites. It is assumed that the ERP termination can be spatially determined as the action potential duration to 80% of repolarization under conditions of short-coupled stimulation. A delay of activation of the following paced action potential (activation latency) approximated excitability for each pixel. As a result, local activation delays associated with pathological remodeling, namely post-repolarization refractoriness are addressed (Coronel et al., 2012). The *θ*_*t,CB*_ parameter is used to separate wave fronts temporally. However, in the current framework, wave fronts that converge, i.e., those originating from independent sources are considered the same wave front. Convergence will be considered for wave fronts that are temporally aligned, i.e., wave front collision sites show pAT gradients inferior to *θ*_*t,CB*_. But tissue generally remains in refractoriness for much longer than *θ*_*t,CB*_ in our experience, meaning wave fronts colliding with local pAT gradients superior to *θ*_*t,CB*_ will be separated.

### 4.2 Conclusion

In conclusion, we provide a comprehensive framework for image processing of complex optical mapping signals, including tachyarrhythmias. An action potential upstroke-windowing scheme based on calculations of phase enabled pixel-wise and upstroke-independent identification of pAT events. This approach is highly robust against changing signal morphology, signal noise, changes to the signal baseline and transitional behavior between non-arrhythmic and arrhythmic states. A crucial component of the image processing pipeline furthermore identifies the spatial organization of pAT points and groups them in to individual wave fronts. A series of analytical and visualization tools permit detailed characterization in a beat-to-beat basis, irrespective of signal complexity.

## Data Availability

The raw data supporting the conclusion of this article will be made available by the authors, without undue reservation.

## Abbreviations

AT, Activation time; APD’min, Minimum action potential duration limit; APD’max, Maximum action potential duration limit; APD’’min, Refined minimum action potential duration limit; APD’’max, Refined maximum action potential duration limit; eAT, Electrical activation time; ERP, Effective refractory period; ERPm*in*, Minimum effective refractory period; F, Fluorescenceπ, Constant Pi; σ_L_, Longitudinal conductivity; T, Number of frames; φ, Phase; θ(*dF*⁄*dt*), Signal derivative threshold; θ_*dF*⁄*dt*_, Signal derivative threshold after outlier removal; *θ*’’_*t,CB*_, Conduction block parameter; pAT, Phase window activation time; RT, Repolarization time; SNR, Signal to noise ratio; t, Time; σ_S_, Transmural conductivity; σ_T_, Transverse conductivity; uAT, Unified window activation time; X, Horizontal image dimension; Y, Vertical image dimension.
