# Supplementary material for: A comprehensive framework for evaluation of high pacing frequency and arrhythmic optical mapping signals
Source: Front Physiol. 2023 Jan 23;14:734356. doi: 10.3389/fphys.2023.734356 (PMC9901579; doi:10.3389/fphys.2023.734356)
Supplement: Supplementary file 1 [file DataSheet1.PDF]

Supplemental materials

Psuedocode for mapping wave front repolarization characteristics

Assuming N AT points in pixel x,y, with T denoting the duration of the movie:

1) **if** $n=N-1$ **then**

    if $AT(x,y,n) + APD'max > T$ **then**

        $APD''max(x,y,n) = T - AT(x,y,n) - 1$

2) **if** $n=N-2$ **then**

    if $AT(x,y,n) + APD'max > \mathrm{T}$ **then**

        $APD''max(x,y,n) = T - AT(x,y,n) - 1$

    else if $AT(x,y,n) + APD'min > AT(x,y,n)$ **then**

        go to $N-1$

    else if $AT(x,y,n) + APD'max > AT(x,y,n)$ **then**

        $APD''max(x,y,n) = AT(x,y,n) - AT(x,y,n) - 1$

3) **if** $n < N-2$ **then**

    if $AT(x,y,n) + APD'max > AT(x,y,n+2)$ **AND** $AT(x,y,n) + ERPmin > AT(x,y,n+2)$ **then**

        $APD''max(x,y,n) = AT(x,y,n+2) - AT(x,y,n) - 1$

    else if $AT(x,y,n) + APD'min > AT(x,y,n+1)$ **then**

        go to $n+1$

    else if $AT(x,y,n) + APD'max > AT(x,y,n+1)$ **then**

        $APD''max(x,y,n) = AT(x,y,n+1) - AT(x,y,n) - 1$

Psuedocode for classification of repetitive and non-repetitive wave fronts

The classification algorithm was implemented using the labelled AT matrix as input where AT points are non-zero, as follows:

for pixel in row x **do**
    for pixel in column y **do**
        for *label* in *labels* **do**
            $N_{AT}(x,y) = \sum_{label} AT(x,y)$
        if $N_{AT}(x,y) > 1$ **then**
            $\boldsymbol{dt_{AT} = (T_{AT,label} + 1) - T_{AT,label}}$
            for *delay* in $n_{Reentrant}(x,y)$ *delays* **do**
                if $delay > ERP_{min}$ **then**
                    $N_{Repetitive}(x,y) = N_{Repetitive}(x,y) + n_{Repetitive}(x,y)$
        else if $N_{AT,Label}(x,y) = 1$ **then**

$$\boldsymbol{N_{Non-repetitive}(x,y) = N_{Non-repetitive}(x,y) + \Big(N_{AT,Label}(x,y) - n_{Repetitive}(x,y)\Big)}$$

If $n_{Repetitive}(x,y) > 1$ **and** $N_{Repetitive}(x,y) = 0$ **then**

$$N_{Non-repetitive}(x,y) = N_{Non-repetitive}(x,y) + n_{Repetitive}(x,y)$$

for *label* in *labels* **do**

if $N_{Repetitive,label} \geq 1$ **then**

$N_{t,repetitive} = \sum_{label} N_{Repetitive}(t)$

DF = **call** find_dominant_frequency($N_{t,reepetitive}$)

N_{rot} = **integer**(DF)

if $Nrot = 1$ **then**

$Class_{repetitive,\ label}$ = False

else

$Class_{repetitive,\ label}$ = True

else $Class_{repetitive,\ label}$ = False

Supplemental figures

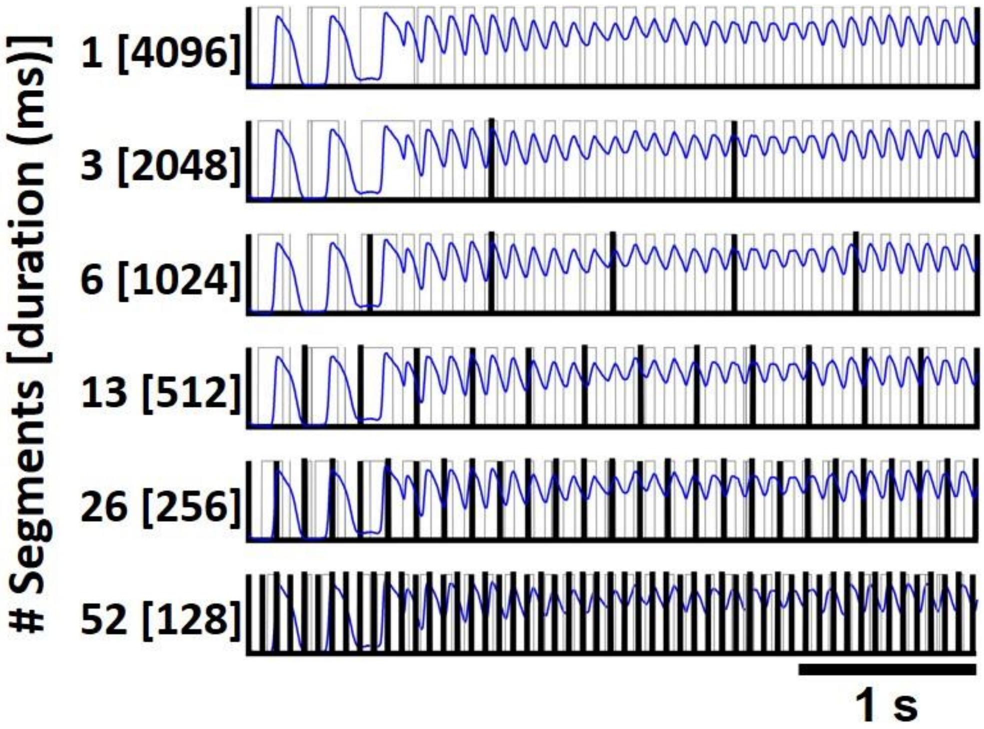

Supplemental figure 1. Signal segmentation for phase-derived upstroke windowing. Given that the Hilbert transform is sensitive to non-periodic signals, which can impact optical mapping recordings during phases of transition between states or complex arrhythmia, signals were processed over varying segment lengths. Signals underwent cycles of phase calculation and signal splitting to maximally identify and window optical action potential upstrokes. At each level of signal splitting, all instances of positive phase deflections were retained for determination of AT.

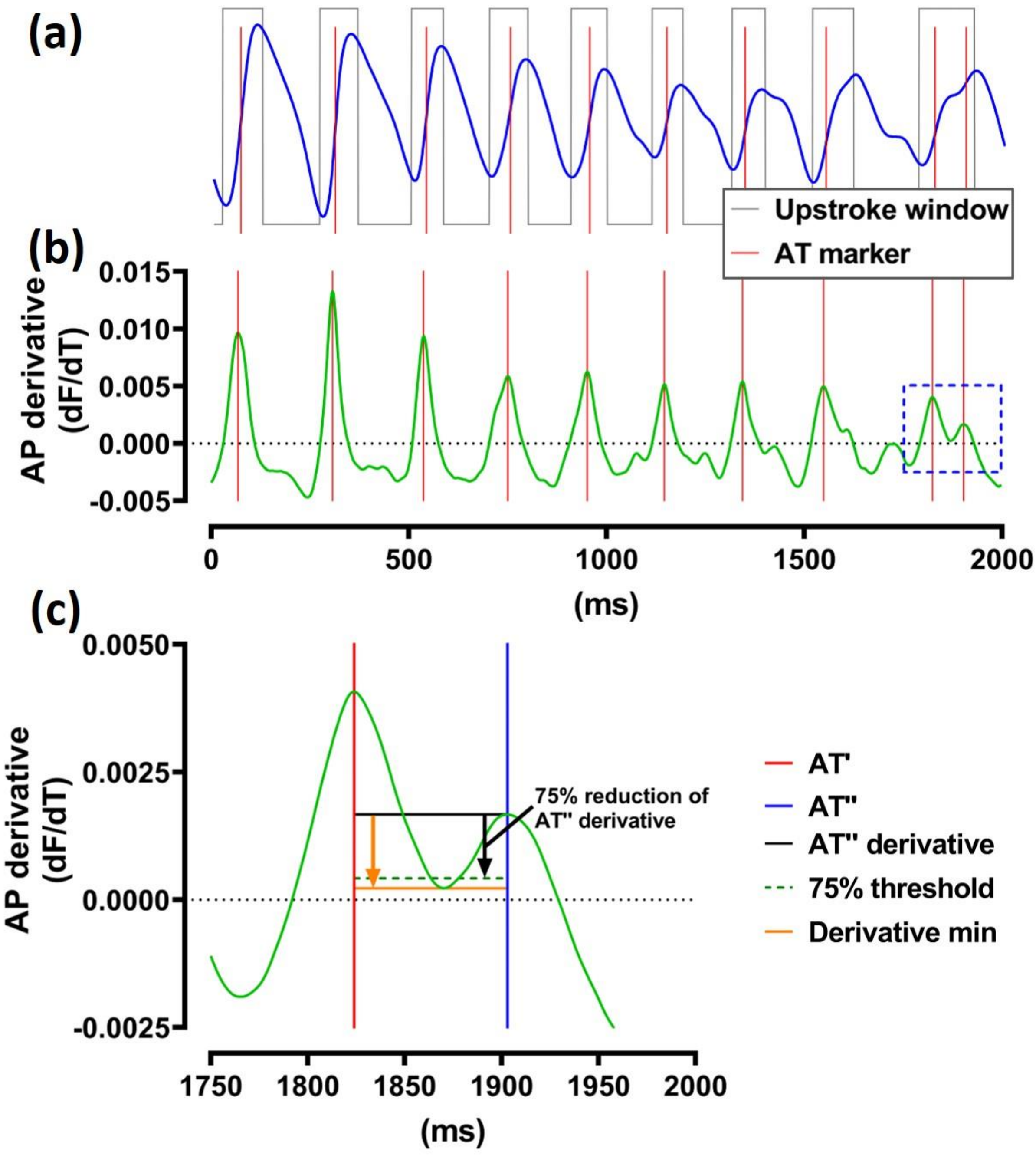

Supplemental figure 2. Phase-derived activation time detection. (a) Optical signal with phase-windows centered on upstrokes. (b) Signal derivative of (a). Derivative peaks corresponding to pAT, indicated by red lines. (c) Double peaks of the signal derivative correspond to a biphasic upstroke morphology. To be eligible for dual activation (pAT at red and blue vertical lines) in a single phase-derived upstroke window, the derivative valley must decrease by 75% from the second largest peak.

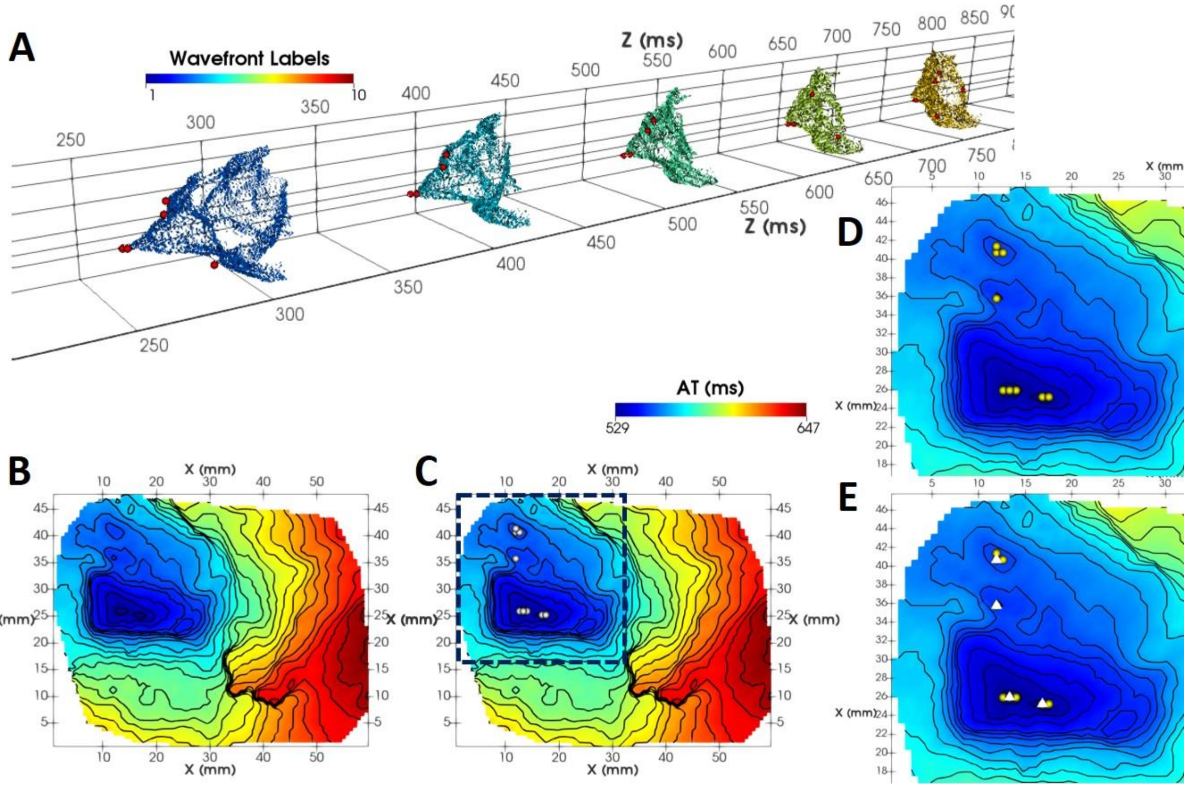

Supplemental figure 3. AT origins. A) A spatial-temporal distribution of wave fronts is shown with AT minima highlighted with red points. **B)** AT map of one wave front. Isochrones are separated by 10 ms. **C)** AT map from B is annotated by yellow points at all pixels corresponding to AT minima. **D)** A blown up image of the dashed box in C. Notice that some minima comprise of multiple pixels. **E)** White triangles indicate the center-most pixel of AT origins.

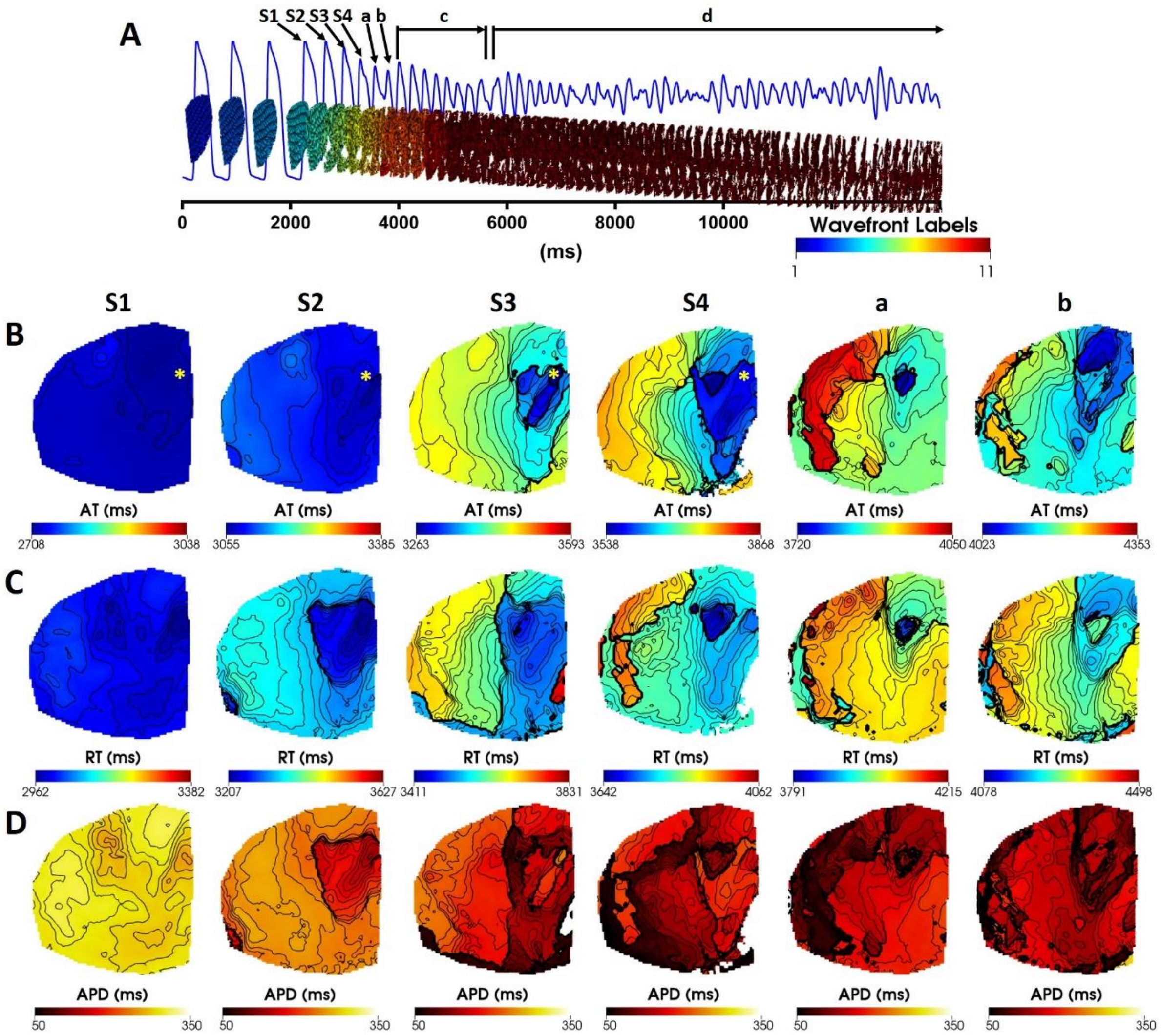

Supplemental figure 4. AT, APD and RT mapping. A) A spatial-temporal distribution of wave fronts during VF induction by S1S2S3S4 pacing. **B)** AT maps corresponding to individual wave fronts elicited by the last of a train of S1 pulses, each short-coupled stimuli and two subsequent autonomously-driven ectopic beats (a and b). **C)** Repolarization time maps corresponding to AT maps from B. **D)** Similarly, action potential duration maps corresponding to maps shown in B. All color maps are scaled to the largest total dispersion across each type of map. Isochrones and isobars are all separated by 10 ms.

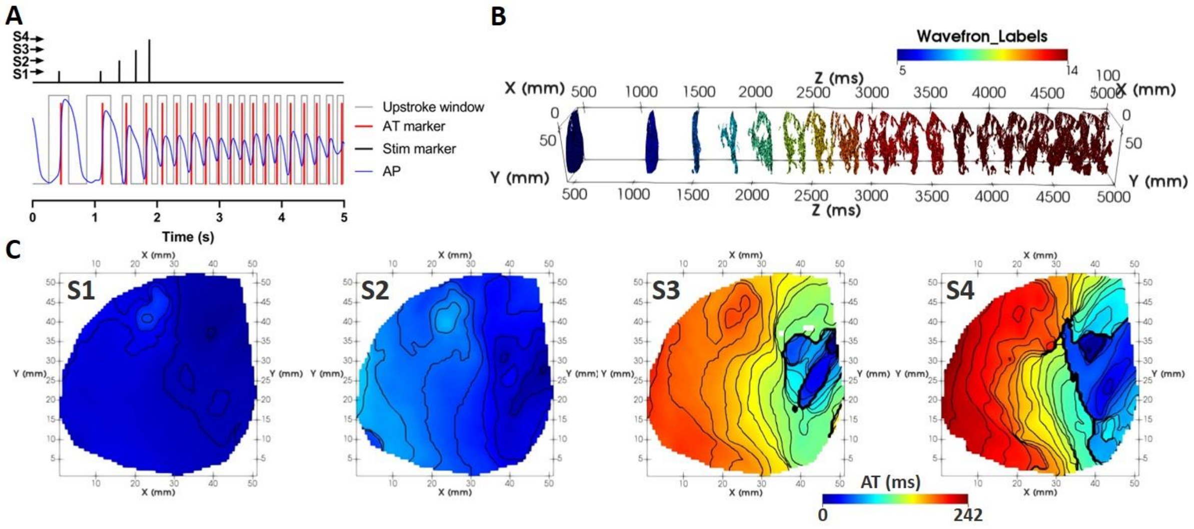

Supplemental figure 5. Case #1 of reproducibility tests for the phase window-derived AT mapping framework. A) An example pre-processed optical action potential trace overlaid with phase-windows centered on upstrokes and markers of ATs. Stimulation markers are shown above. Total acquisition length analyzed was 16126 ms in length. **B)** A spatial-temporal distribution of labelled wave fronts. A total of 40 wave fronts were identified. 11 beats out of 51 were repetitive. **C)** AT maps corresponding to each level of stimulation. Color maps have each been scaled to the same AT scale. Isochrones are separated by 10 ms.

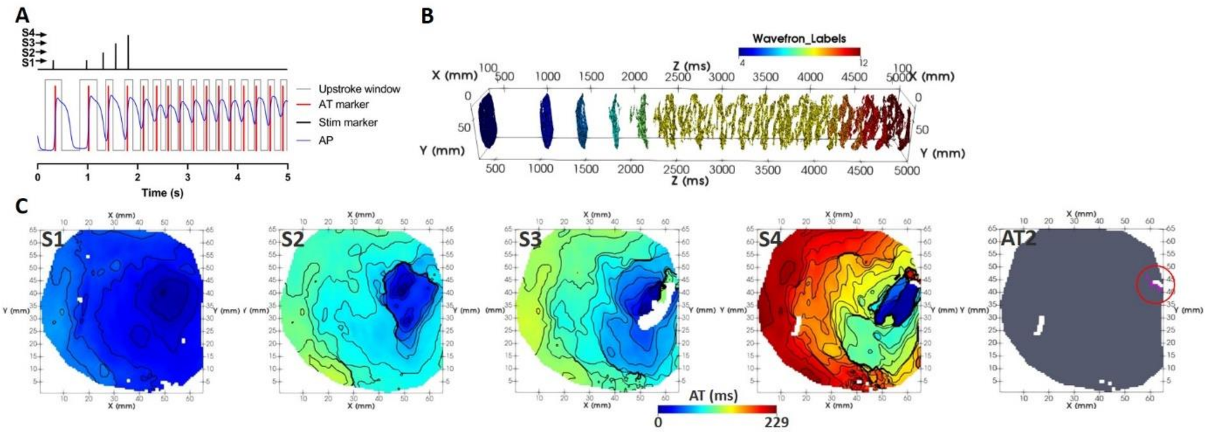

Supplemental figure 6. Case #2 of reproducibility tests for the phase window-derived AT mapping framework. A) An example pre-processed optical action potential trace overlaid with phase-windows centered on upstrokes and markers of ATs. Stimulation markers are shown above. Total acquisition length was 6525 ms. **B)** A spatial-temporal distribution of labelled wave fronts. A total of 7 wave fronts were identified. 15 out of 21 beats were repetitive. **C)** AT maps corresponding to each level of stimulation. Color maps have each been scaled to the same AT scale. Isochrones are separated by 10 ms. A separate map, AT2, indicates the locations where dual upstroke morphologies were identified (highlighted by a red circle).

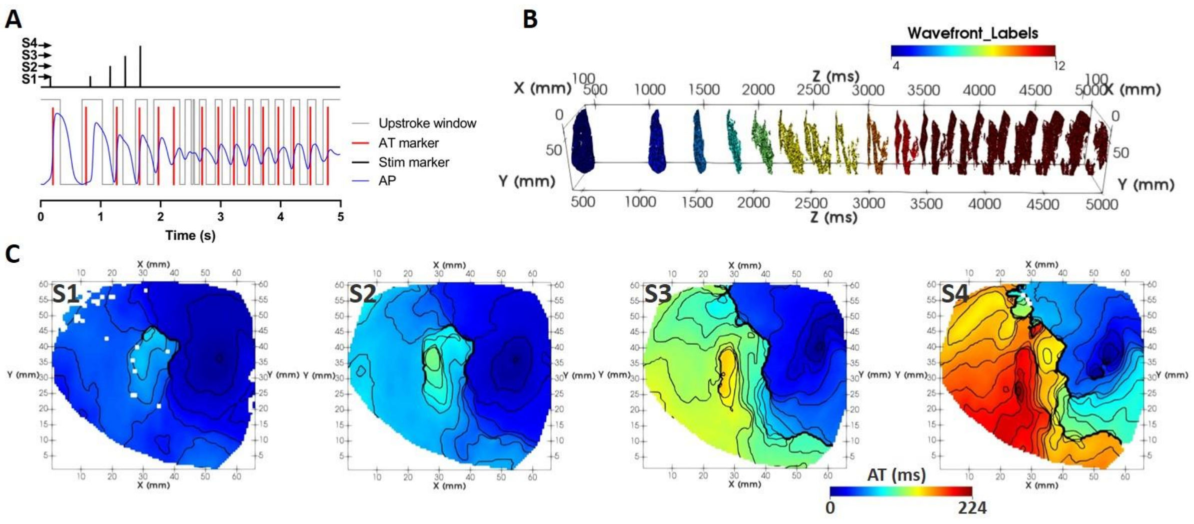

Supplemental figure 7. Case #3 of reproducibility tests for the phase window-derived AT mapping framework. A) An example pre-processed optical action potential trace overlaid with phase-windows centered on upstrokes and markers of ATs. Stimulation markers are shown above. Total acquisition length analyzed was 15298 ms in length. **B)** A spatial-temporal distribution of labelled wave fronts. A total of 10 wave fronts were identified. 47 beats out of 57 were repetitive. **C)** AT maps corresponding to each level of stimulation. Color maps have each been scaled to the same AT scale. Isochrones are separated by 10 ms.

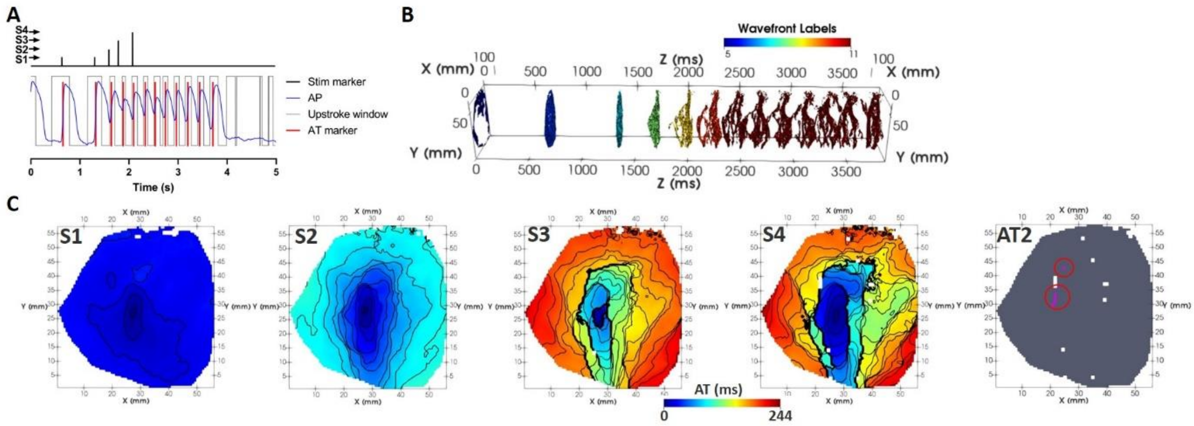

Supplemental figure 8. Case #4 of reproducibility tests for the phase window-derived AT mapping framework. A) An example pre-processed optical action potential trace overlaid with phase-windows centered on upstrokes and markers of ATs. Stimulation markers are shown above. Total acquisition length analyzed was 6840 ms in length. **B)** A spatial-temporal distribution of labelled wave fronts. A total of 1 wave fronts were identified. 9 beats were repetitive. **C)** AT maps corresponding to each level of stimulation. Color maps have each been scaled to the same AT scale. Isochrones are separated by 10 ms. A separate map, AT2, indicates the locations where dual upstroke morphologies were identified (highlighted by a red circle).

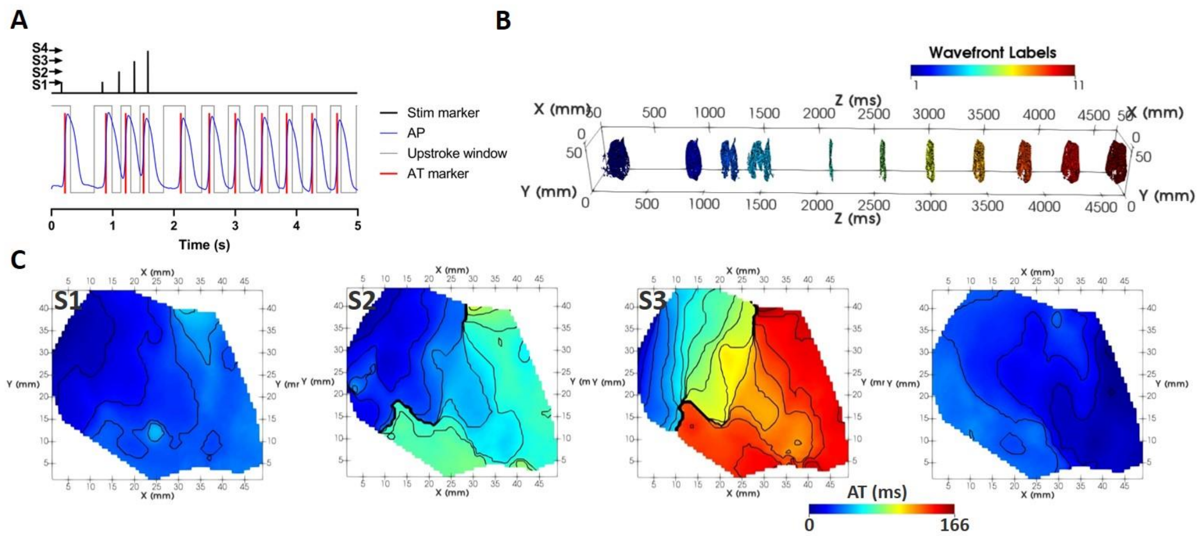

Supplemental figure 9. Case #5 of reproducibility tests for the phase window-derived AT mapping framework. A) An example pre-processed optical action potential trace overlaid with phase-windows centered on upstrokes and markers of ATs. Stimulation markers are shown above. Total acquisition length analyzed was 6840 ms in length. **B)** A spatial-temporal distribution of labelled wave fronts. A total of 40 wave fronts were identified. 11 beats out of 51 were repetitive. **C)** AT maps corresponding to each level of stimulation. Color maps have each been scaled to the same AT scale. Isochrones are separated by 10 ms.

An example pre-processed optical action potential trace overlaid with phase-windows centered on upstrokes and markers of ATs. Stimulation markers are shown above. Total acquisition length analyzed was 7924 ms in length. **B)** A spatial-temporal distribution of labelled wave fronts. A total of 40 wave fronts were identified. 6 beats out of 12 were repetitive. **C)** AT maps corresponding to each level of stimulation. Color maps have each been scaled to the same AT scale. Isochrones are separated by 10 ms.

Supplemental videos

Supplemental video 1. Phase mapping reentry.
